# Analysis of the Phospholipid Profile of the Collection Strain PAO1 and Clinical Isolates of *Pseudomonas aeruginosa* in Relation to Their Attachment Capacity

**DOI:** 10.3390/ijms22084003

**Published:** 2021-04-13

**Authors:** Caroline Le Sénéchal, Mathilde Puges, Christophe Barthe, Patricia Costaglioli, Caroline Tokarski, Corinne Buré, Sébastien Vilain

**Affiliations:** 1 CNRS, Bordeaux INP, CBMN, University Bordeaux, UMR 5248, F-33600 Pessac, France; caroline.le-senechal@u-bordeaux.fr (C.L.S.); christophe.barthe@u-bordeaux.fr (C.B.); patricia.costaglioli@bordeaux-inp.fr (P.C.); caroline.tokarski@u-bordeaux.fr (C.T.); c.bure@cbmn.u-bordeaux.fr (C.B.); 2 Infectious and Tropical Diseases Department, CHU of Bordeaux, F-33000 Bordeaux, France; mathilde.puges@u-bordeaux.fr

**Keywords:** *Pseudomonas aeruginosa*, cystic fibrosis, clinical strain, biofilm, phospholipid, lipidomics, mass spectrometry

## Abstract

Bacteria form multicellular and resistant structures named biofilms. Biofilm formation starts with the attachment phase, and the molecular actors involved in this phase, except adhesins, are poorly characterized. There is growing evidence that phospholipids are more than simple structural bricks. They are involved in bacterial adaptive physiology, but little is known about their role in biofilm formation. Here, we report a mass spectrometry analysis of the phospholipid (PL) profile of several strains of *Pseudomonas aeruginosa* isolated from cystic fibrosis patients. The aim of our study was to evaluate a possible link between the PL profile of a strain and its attachment phenotype. Our results showed that PL profile is strongly strain-dependent. The PL profile of *P. aeruginosa* PAO1, a collection strain, was different from those of 10 clinical isolates characterized either by a very low or a very high attachment capacity. We observed also that the clinical strain’s PL profiles varied even more importantly between isolates. By comparing groups of strains having similar attachment capacities, we identified one PL, PE 18:1-18:1, as a potential molecular actor involved in attachment, the first step in biofilm formation. This PL represents a possible target in the fight against biofilms.

## 1. Introduction

*Pseudomonas aeruginosa* is an opportunistic pathogen able to adapt to numerous environments. This bacteria belongs to the ESKAPE group of pathogens that exhibit multidrug resistance and virulence [1]. It is classified by the World Health Organization among the top three “critical pathogens” for which new antibiotics are urgently needed and research is encouraged to decipher the mechanisms involved in antibiotic recalcitrance [2,3]. The ability to form biofilms is part of recalcitrance [3]. A biofilm is a surface-associated microbial community composed of microcolonies embedded in a self-produced extracellular polymeric substance, named a matrix. Bacteria organized in biofilms, called sessile bacteria, are characterized by exceptional resistance to many stresses and biocides, in particular antibiotics, compared to their planktonic counterparts [4]. Research in biofilmology is very active because the exact origins of recalcitrance are poorly understood to date. Although having positive applications in biotechnology [5], biofilms are the source of many problems in industrial settings or on medical materials. Combating their formation is a necessity from both an economic and a public health point of view [6].

Strains of *P. aeruginosa* from collections, such as PAO1, are reference organisms extensively used to decipher the mechanisms involved in virulence, resistance and biofilm formation [7]. *P. aeruginosa* strains are involved in several infections including wound, burn, urinary tract, gastrointestinal and hospital-acquired infections [8] and form biofilms in the lungs of cystic fibrosis patients, leading to impaired lung function as well as increased morbidity and mortality [9]. To fight against biofilms, it is essential to decipher the physiology of sessile cells and identify the molecular actors involved in their development. In this aim, numerous studies were performed, but they often used different bacterial strains, systems, nutritional sources and growth conditions, among others, leading to great disparities in results [10,11]. A common approach to identify potential molecular targets is to compare the molecular content, via a “omic” technology for instance, of the sessile cells of a collection strain immobilized on a biotic or abiotic surface to the molecular content of the planktonic cells of the same strain, sometime cultivated in a different system than the one used to obtain the biofilms [12]. This approach is potent but may show limitations. On the one hand, the strain is often well-characterized, especially at the genomic level, and is studied in a controlled environment, but, on the other hand, the use of a collection strain, domesticated by intensive use in the laboratory, studied in oversimplified experimental conditions, produces results far from in vivo conditions and from which it is impossible to distinguish a cause from a consequence of the biofilm mode of growth [13].

Our work aims to identify the molecular actors involved in the attachment phase, the first step in the formation of a biofilm. In this context, we developed an original system of immobilization, named GW2S, allowing us to obtain an important immobilized biomass in a very short time [14]. Following a proteomic study [15], we analyzed the phospholipid profiles, named phospholipidomes in the rest of the text, of attached (sessile) and unattached (planktonic) *P. aeruginosa* PAO1 cells incubated in our system. The results indicated that the majority of the modifications in the phospholipid relative amounts occurred between 3 and 6 h of incubation, but some phospholipid species presented statistically different amounts in attached cells compared to the unattached ones after a few minutes of incubation [16]. To complete these results and enhance our experimental strategy to identify new molecular targets, we built up a library of *P. aeruginosa* clinical strains from patients with cystic fibrosis. The lungs of the patients affected by this genetic disease are congested with viscous mucus, which, in fine, is often colonized by *P. aeruginosa* biofilms, the leading cause of death in cystic fibrosis [17,18]. The ability of each clinical isolate to colonize our GW2S system was determined and 10 clinical strains with extreme and opposite attachment capacities were selected: five characterized by a very low attachment capacity and five by a very high one. We report here the analysis of phospholipid profiles of these 10 clinical isolates, their comparison with the phospholipidome of *P. aeruginosa* PAO1, and a possible link to the strain’s attachment capacities is discussed.

## 2. Materials and Methods

### Isolation, Identification and Growth Conditions of Bacterial Strains

A *Pseudomonas aeruginosa* PAO1 strain (CIP 104116) was obtained from the Institut Pasteur (Paris). Clinical strains of *P. aeruginosa* were isolated from cystic fibrosis adult and underage patients’ sputa, obtained at the University Hospital of Bordeaux, with patients’ informed consent. Each sputum was treated with Sputolysin (1V/1V) (Calbiochem) for 20 min at 4 °C. Then, 100 µL was plated on lysogeny broth (LB) agar (LBA) (tryptone 10 g/L, yeast extract 5 g/L, NaCl 5 g/L, agar 15 g/L). After 18 h at 37 °C, colonies from a quarter of the plate were suspended in 1 mL of PBS (NaCl 8 g/L, KCl 0.2 g/L, Na_2_HPO_4_ 2H_2_O 1.44 g/L, KH_2_PO_4_ 0.24 g/L) and 100 µL of serial dilutions (10^−1^ to 10^−7^) were spread out on cetrimide selective agar (Sigma-Aldrich). After 18 h at 37 °C, selected colonies were suspended in LB/glycerol (1 V/1 V) and stocked at −80 °C. The identification of selected colonies was performed by mass spectrometry. For each isolate, a small part of one colony was spotted onto a 96-spot steel plate (Bruker Daltonics, Bremen, Germany) and allowed to dry at room temperature before the addition of 1 μL of the HCCA matrix (α-cyano-4-hydroxycinnamic acid) provided by the supplier. The spots were crystallized by air-drying before the addition of 1 μL of 70% formic acid. Each sample was tested in duplicate, but only the spot returning the highest probability score of identification was considered. The protein mass spectra were analyzed using Flex Control software and the MALDI Biotyper version 3.1 7311 reference spectra (Bruker Daltonics, Bremen, Germany). The MALDI-TOF MS results were obtained according to the manufacturer’s technical specifications as follows: correct genus and species identification (≥2.0), correct genus identification (1.7–2.0) and no reliable identification (<1.7). All samples had a score >2. The growth conditions for all experiments were identical. Strains were plated on LBA from the −80 °C stock and grown for 18 h at 37 °C. The colonies from 1 LBA plate were then suspended in LB (pH 7.2) and bacterial suspensions, used as inoculum, were calibrated at ≈10^7^ CFU/mL.

## 3. Attachment Test

Based on our previously described method [14], the attachment capacity (AC) on glass wool (GW) of *P. aeruginosa* PAO1 and of 159 clinical strains was determined in order to select strains exhibiting extreme AC (very low or very high). We defined the AC as the proportion of bacterial cells from the inoculum that adhere to the GW fibers after 20 min of incubation. Thus, an AC of 40% means that, out of 100 cells in contact with GW, 40 cells strongly adhered to the fiber after 20 min while 60 cells did not adhere or adhered so weakly that they were flushed away during the washing step. All of the numerical values of AC are indicated rounded to the nearest unit. For each strain, 5 mL of a calibrated bacterial suspension (≈10^7^ CFU/mL) was adsorbed on a 1 g piece of GW and incubated for 20 min at 37 °C under agitation (150 rpm). Then the piece of GW was placed in a 50 mL syringe and unattached cells were harvested by running down 100 mL of PBS through the GW by gravity. Afterwards, the GW piece was placed in 100 mL PBS and attached cells were harvested from the GW by vortexing vigorously for 30 s. The unattached or attached cells contained in PBS solutions were quantified by colony-forming-unit (CFU) counting by plating 100 µL aliquots of serial dilutions twice onto LBA and reading the plates after 24 h at 37 °C. All AC tests were performed in biological triplicate (*n* = 3), and rounded values are presented as mean ± SD.

## 4. Phospholipids Extraction

Phospholipids (PLs) from the 10 selected clinical strains and *P. aeruginosa* PAO1 were extracted according to a previously described method [16]. For each strain, eight PL extractions were performed from eight biological replicates (*n* = 8). PLs were extracted from the cells by mixing 180 µL of the calibrated bacterial suspension to 990 μL of CHCl_3_/CH_3_OH 17:1 (*v*/*v*) in order to eliminate water-soluble metabolites and proteins. The resulting lysate was incubated for 2 h at 4 °C under agitation (1400 rpm). The aqueous phase was harvested after centrifugation (2 min at 4 °C at 13.3 g) and the PLs were re-extracted by adding 990 μL of CHCl_3_/CH_3_OH 2:1 (*v*/*v*). The mix was incubated for 1 h at 4 °C under agitation (1400 rpm). The polar lipids were isolated in the organic phase after centrifugation (2 min at 4 °C at 13.3 g), and the PL extract was dried under vacuum.

## 5. Mass Spectrometry Analyses of *Pseudomonas Aeruginosa* Phospholipids

### 5.1. Identification of PL by Shotgun Mass Spectrometry

Initial identification of PLs was previously performed from PAO1 cells by shotgun-mass-spectrometry analysis using electrospray ionization (ESI) [19]. Briefly, the dried PL extract was suspended in 300 µL CHCl_3_/CH_3_OH 2:1 (*v*/*v*) containing 8 mM ammonium acetate and then was diluted 50 times in a mixture of chloroform/methanol 2/1 (*v*/*v*) containing 8 mM ammonium acetate. The sample was infused into the TurboV electrospray source of a QTRAP 5500 mass spectrometer (Sciex, Villebon sur Yvette, France) at a flow rate of 7 µL/min, with fast polarity switching (50 ms) at a scan rate of 200 Da/s. ESI-MS/MS experiments in precursor ion scan were performed in negative ion mode to screen for phosphatidylethanolamines (PE), phosphatidylserines (PS), phosphatidic acids (PA), phosphatidylinositols (PI) and phosphatidylglycerols (PG); this process led to 54 precursor ion scans. In positive ion mode, one precursor ion scan allowed screening for phosphatidylcholines (PC). PL species were identified using Lipid View software (v1.0, Sciex).

### 5.2. Relative Quantification of PLs by Reverse-Phase Liquid Chromatography-Mass Spectrometry (RPLC-MS/MS)

PLs were quantified by reverse-phase liquid chromatography-tandem mass spectrometry. PL dried extracts were suspended in 300 µL isopropanol/methanol/water 50/10/40 (*v*/*v*/*v*) and then were diluted 20 times in the sample solvent for injection. A solution of external standards composed of PE 17:0-17:0, PC 17:0-17:0 and PG 17:0-17:0 at 0.5, 0.05 and 0.5 μM concentration, respectively (Avanti Polar Lipids, Alabaster, AL, USA) in isopropanol/methanol/water 50/10/40 (*v*/*v*/*v*) was injected separately from the sample. RPLC was carried out at 40 °C on a Luna 5u C8, with 100A pore size, 150 × 1 mm and 5 µm particles (Phenomenex, Le Pecq, France). The gradient elution program was a combination of eluent A (isopropanol/methanol/water 50/10/40 (*v*/*v*/*v*) with 0.2% formic acid and 0.028% ammonium hydroxide) and eluent B (isopropanol with 0.2% formic acid and 0.028% ammonium hydroxide) with 30% B (0 min); 50% B (0–5 min); 80% B (5–30 min); 30% B (30–35 min). The flow rate was set at 40 µL/min; 5 µL sample volumes were injected. RPLC-MS/MS analyses were performed with a 5500 QTRAP (Sciex) instrument coupled to an LC system (LC-20AD XR pump, Shimadzu, Marne-la-Vallée, France) and a PAL HTC-xt Autosampler (CTC Analytics, Zwingen, Switzerland). MS analyses were carried out in the negative (PE, PG) and positive (PC) modes with fast polarity switching (50 ms). The RPLC-MS/MS method in multiple reaction monitoring (MRM) mode was adapted from Buré et al. [19]. Nitrogen was used as curtain gas (set to 20), gas1 (set to 25) and gas2 (set to 0). Needle voltage was at −4500 V or +5500 V without needle heating; declustering potential was between −180 V and −85 V or set at +40 V. The collision gas was nitrogen; collision energy varied from 47 to 62 eV on a compound-dependent basis. The dwell time was set to 3 ms. MS/MS experiments were performed by 7 positive MRM scans and 41 negative MRM scans according to the PL identifications obtained by shotgun MS. Area of LC peaks were determined using MultiQuant software (v2.1, Sciex). The peak area of each PL was normalized in comparison to the peak area of the external standard used for the molecular species it belongs to (e.g., PC 17:0/17:0, PE 17:0/17:0 or PG 17:0/17:0), according to a method adapted from Ejsing et al. [20]. The sum of the normalized areas of all PLs belonging to the same molecular class (PC, PE or PG) represents 100% of this molecular class.

## 6. Statistical Analysis

All statistical tests were performed using Statgraphic Plus 5.1 software (StatPoint Technologies Inc., Warrenton, VA, USA). The relative standard deviation (RSD) for each PL (*n* = 28) in strain (*n* = 11, i.e., PAO1 + 10 clinical strains) was determined from the data obtained through the biological replicates (*n* = 8 per strain; i.e., octuplate). The 308 RSD so-calculated were used as indicators of the repeatability of the data and charted on a box-and-whisker plot. The nonparametric tests Kruskal–Wallis and Mann–Whitney were used to determine statistically significant differences. A principal component analysis (PCA), a multivariate analysis, was performed with centered reduced values to reveal existing correlations within our data. The PCA results were visualized as a component plot. PCA easily highlights potential correlations among variables (i.e., strain PL profile in our case) using a graphic visualization of correlations either by grouping highly correlated, or separating weakly correlated variables, according to the axes of the principal components. The degree of correlation of the two variables may be also estimated by the angle between vectors representing the original variables and pointing away from the origin of axes. A small angle indicates a positive correlation, an angle of 90° indicates that variables are not correlated, whereas an angle of 180° indicates a negative correlation between two variables.

## 7. Results

### Attachment Capacities and Selection of P. aeruginosa PAO1 and Clinical Strains

**Figure 1:**
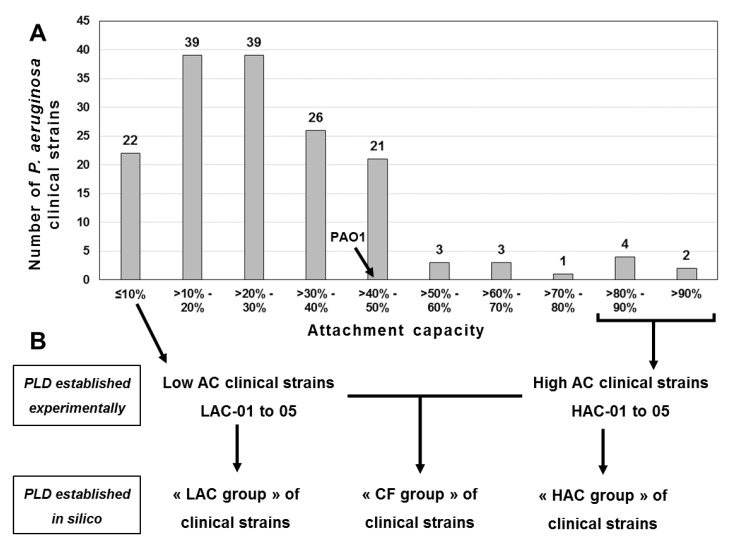
Attachment capacities of a collection and clinical *Pseudomonas aeruginosa* strains. (**A**) The attachment capacity (**AC**) of each *P. aeruginosa* strain was determined using a system initially developed to study the attachment phase, the first step in biofilm formation [14]. This system uses glass wool fibers as a colonization surface (see the *Material and Methods* section). We defined the AC as the proportion of bacterial cells from an inoculum that adhered to the glass wool fibers after 20 min of incubation. This value is expressed as a percentage and is rounded to the nearest unit. The phospholipidomes (PLDs) of *P. aeruginosa* PAO1, a collection strain, and of 10 cystic fibrosis (**CF**) clinical strains, were obtained from octuplates (*n* = 8) of each strain. (**B**) The CF strains selected for this study comprised the five isolates exhibiting the lowest attachment capacities (**LAC-01 to -05**) and the five isolates with the highest attachment capacities (**HAC-01 to -05**). In silico PLDs were determined from experimental data obtained for all CF strains (**CF group**), LAC strains (**LAC group**) or HAC strains (**HAC group**).

From 19 adult and 14 underage cystic fibrosis (CF) patients, 33 sputa were collected, from which 159 clinical strains were isolated on cetrimide agar, a selective medium of *Pseudomonas aeruginosa* strains. After identification by mass spectrometry, the attachment capacity (AC) of each *P. aeruginosa* clinical strain was determined using our GW2S system, initially developed to study the attachment phase [14], the first step in biofilm formation. We defined the AC as the proportion of bacterial cells from an inoculum that adheres to the GW fibers after 20 min of incubation. This value is expressed as a percentage. The distribution of strains according to their AC is shown in Figure 1. The average AC of the collection strain PAO1 was estimated to be 41 ± 3%. Regarding the clinical isolates, the AC values were highly variable, ranging from ≈1% to ≈91%, and not uniformly distributed as indicated by the quartiles of the distribution: first quartile ≈ 15%, median ≈ 25%, 3rd quartile ≈ 39%. Indeed, out of the 159 clinical strains, 147 (92%) had an AC less than 50%, while 128 (81%) strains exhibited an AC lower than PAO1, and only 31 (19%) had an AC higher than that of the reference strain. At the extremes of the distribution, 22 clinical isolates were characterized by low values of AC (≤10%) and 6 by high values (>80%), of whom 2 had the highest AC (>90%) (Figure 1A). To study the attachment phase, the five strains with the lowest AC values (<4%) and the five strains with the highest AC values (>85%) were selected. According to ascending AC values, the strains with «low attachment capacity» (LAC) were named LAC-01 (1 ± 0%), LAC-02 (2 ± 0%), LAC-03 (2 ± 0%), LAC-04 (2 ± 1%) and LAC-05 (3 ± 0%) and the strains with «high attachment capacity» (HAC) were named HAC-05 (85 ± 1%), HAC-04 (87 ± 1%), HAC-03 (89 ± 2%), HAC-02 (90 ± 3%) and HAC-01 (91 ± 1%). These 10 clinical isolates represent the so-called “CF group” of strains in this publication. This group is composed of two subgroups: the “LAC group”, clustering the five LAC strains, and the “HAC group”, composed of the five HAC strains (Figure 1B). All of these strains were isolated from adult patients’ sputa; HAC isolates were from two different sputa, whereas LACs were from three other distinct sputa.

## 8. Phospholipidome Differences between *P. aeruginosa* PAO1 and Clinical Strains

**Figure 2:**
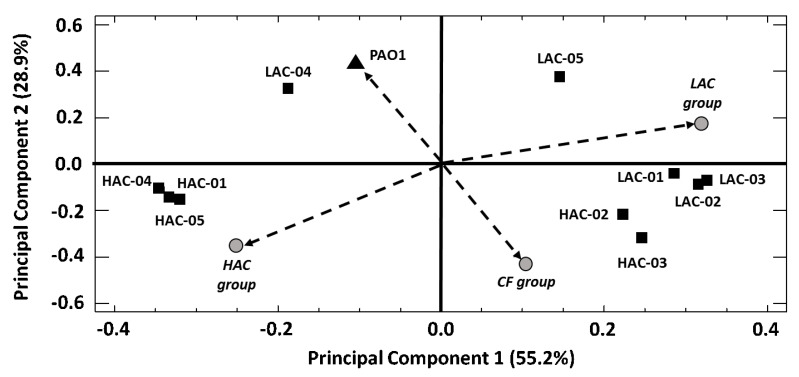
Principal component analysis (PCA) of the experimental and in silico phospholipidomes of the *Pseudomonas aeruginosa* strains. The mean experimental phospholipidome (filled black symbols) of *Pseudomonas aeruginosa* PAO1, a collection strain (▲), was calculated from eight biological replicates. In a similar manner, phospholipidomes of 10 cystic fibrosis (CF) clinical strains (■) were obtained for the 5 CF strains exhibiting the lowest attachment capacities (LAC-01 to -05) and the 5 CF strains with the highest attachment capacities (HAC-01 to -05). In silico phospholipidomes (filled gray circles) were determined from experimental data obtained for all CF strains (CF group), LAC strains (LAC group) or HAC strains (HAC group). The phospholipids were quantified as detailed in the *Materials and Methods* section. The component plot was obtained via Statgraphic Plus 5.1 software using the PCA matrix presented in Table S1 (see “PCA”).

The phospholipid (PL) profiles, named phospholipidome (PLD), of the 10 selected clinical strains and the collection strain PAO1 were determined experimentally by liquid chromatography/tandem mass spectrometry. Phosphatidylserines, phosphatidic acids and phosphatidylinositols being not detected, only phosphatidylcholine (PC), phosphatidylethanolamine (PE) and phosphatidylglycerol (PG) composed the PLD [16]. From these data, in silico PLDs were established for the “CF group” as well as the “LAC” and “HAC” groups (Figure 1B). In silico PLD was calculated by averaging, for each PL, the experimental values of all the strains included in a group. In our study, 28 molecular species of PL were quantified in each *P. aeruginosa* strain, belonging to the PC, PE or PG classes of PL (Table S1: “PL data” sheet). In the PAO1 strain, the main PC species were PC 34:1, PC 35:1 and PC 34:2, which represented, respectively, 55.3% ± 1.5%, 12.9% ± 1.6% and 11.3% ± 0.8% of the 7 quantified PCs. The main PE species of the 12 quantified were PE 16:0-18:1 (43.2% ± 2.4%), PE 16:0-19:1 (16.0% ± 1.8%) and PE 16:1-18:1 (13.4% ± 1.9%), whereas among the 9 quantified PGs, PG 16:1-18:1 (41.6% ± 4.5%), PG 16:0-19:1 (26.4% ± 4.5%) and PG 17:1-18:1 (9.3% ± 0.9%) were the most abundant. In order to compare in a practical way the PLD of PAO1 with those of the clinical strains, a principal component analysis (PCA) was performed with all PLDs (in silico and experimental) (Table S1: “PCA” sheet). PCA is a multivariate analysis that easily highlights the correlations among variables (e.g., PLD) by graphic visualization. Here, the PCA extracted two principal components, collectively accounting for 84.1% of the data variability that clearly segregated PAO1 and all in silico PLDs (i.e., “CF”, “LAC” and “HAC” groups) (Figure 2). In particular, the vectors of PAO1 and the “CF group” formed an angle of 180°, whereas the PAO1 vector formed angles closer to 90° with both the “LAC group” and “HAC group” vectors (cf. *Materials and Methods* section for precision). This distribution of PLDs indicated major differences between the PL profile of PAO1 and those of the clinical strains (Figure 2). For the sake of clarity, we present here the comparison between the PLDs of PAO1 and the “CF group” and very briefly discuss the comparison with the “LAC” or “HAC” groups with a specific clinical strain. All the data allowing comparisons of PAO1 with the “LAC” and “HAC” groups, as well as those of each clinical strain, are available in Supplementary data (Table S1: “PAO1 vs. Clinical strains” sheet). To identify the main differences distinguishing the PLD of PAO1 from the “CF group”, we compared the data for each PL and focused on the PLs fulfilling the following criteria: a minimum factor of two between the mean relative quantities and a statistical difference between the data (*p*-value < 0.05, Mann–Whitney test). Additionally, the rank of a PL is discussed. The rank of a PL is linked to its relative abundance within a PL class. For instance, the rank of PC 34:1 is 1 in 7 because its relative quantity is the greatest of the seven quantified PCs (Table 1 and Table S1: “Means & SD” sheet). Out of the 28 quantified PL, 19 presented a statistical difference between the relative quantities of “CF group” and PAO1, but only 9 presented with a factor ≥ 2 (Table 2 and Table S1: “PAO1 vs. Clinical strains” sheet). Among the seven PC species quantified, the mean relative quantity of PC 32:0 was 2.5 times higher, whereas those of PC 35:1 and PC 35:2 were, respectively, 3.0 and 2.2 times less important in the “CF group” compared to PAO1. This modification of the relative quantity led to variation in the two first PCs’ relative representativeness as the ranking of PC 32:0 increased from the sixth most abundant PCs in the PLD of PAO1 to the third rank in “CF group” one, whereas the ranking of PC 35:1 fall down from the second to the sixth place. These modifications were similar when PAO1 was compared to the “LAC” or “HAC” groups (Table 2 and Table S1: “PAO1 vs Clinical strains” sheet). Among the 12 quantified PE, PE 16:0-16:0 and PE 16:0-16:1 exhibited higher relative quantities (factors of 2.2 and 2.3, respectively) in the “CF group” PLD compared to the PAO1 one, whereas their ranking moved from seventh to third and ninth to fifth rank, respectively. At the opposite end, the relative quantities of PE 16:0-19:1 and PE 18:1-18:1 were, respectively, 2.0 and 3.3 times lower, and their ranks moved from 2nd to 4th and 5th to 10th rank. Except for PE 16:0-19:1, all these differences appeared to be linked to the “HAC group”, which had the relatively biggest changes in PE content compared to the “LAC group”. Finally, the mean ranking of PG 16:0-16:0 in clinical strains was the fourth rank on nine quantified PGs, compared to eighth place in PAO1, due to an increase of its relative quantity by a factor of 2.0 whereas, a contrario, PG 18:0-18:1’s relative quantity was reduced by a factor of 2.2 without changing its ranking (ninth place) (Table 2 and Table S1: “PAO1 vs. Clinical strains” sheet). As for PE, these differences seem to be more marked in the “HAC group”. In a strain-by-strain comparison, considering all values (i.e., 8 replicates/PL × 10 strains vs 8 replicates/PL for PAO1) of the PLs’ relative quantity, we noticed that the modification factor for PL enrichment in a clinical strain compared to PAO1 does not exceed a factor of 5.9, whereas the one for a decrease reached a factor of 28.6. These factors were 3.6 and 15.2, respectively, when considering only the mean values (Table S1: “PAO1 vs. Clinical strains” sheet). Altogether, our results showed that the PLD of *P. aeruginosa* PAO1 is, according to the statistical test, strongly different than the in silico PLDs computed from the PL profiles experimentally determined of the 10 clinical strains. Nevertheless, a deeper analysis indicated that these differences concerned numerous PLs but often with a modest amplitude, in particular in the case of PL enrichment in a clinical strain.

## 9. Differences among the Phospholipidomes of the *P. aeruginosa* Clinical Strains

**Figure 3:**
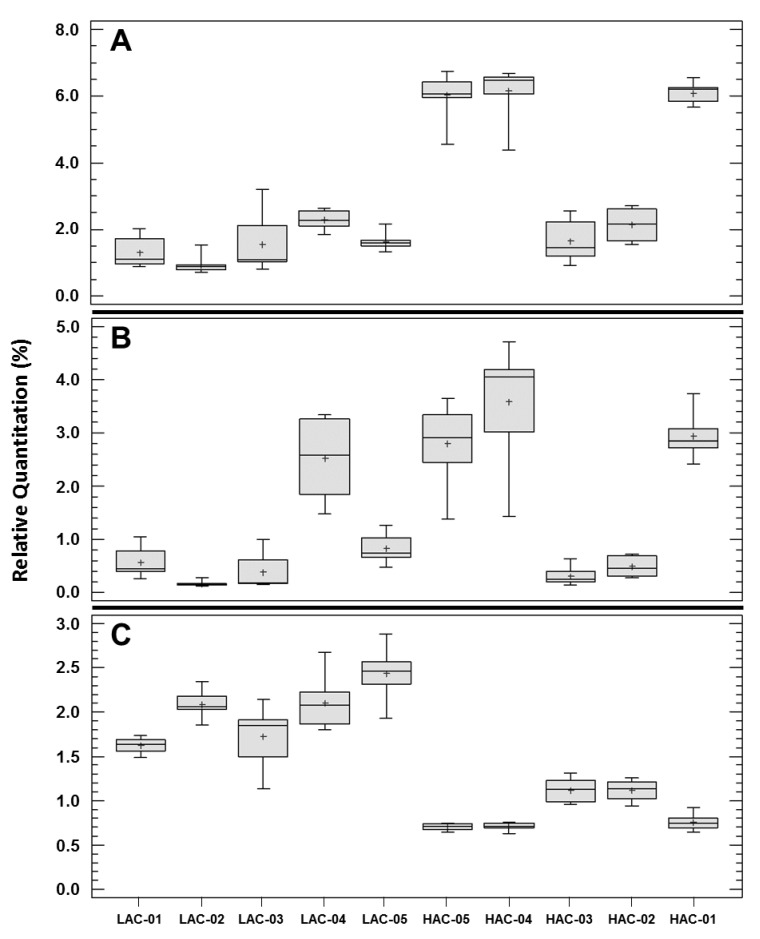
Relative quantitation of 3 phospholipids in clinical strains of *Pseudomonas aeruginosa* exhibiting extreme and opposite attachment capacities. Among the 28 phospholipids quantified through the 11 *P. aeruginosa* strains selected for this study, three phosphatidylethanolamine species, PE 16:0-17:1 (**A**); PE 17:1-19:1 (**B**) and PE 18:1-18:1 (**C**), presented both a statistical difference and a factor >2 between the mean percentage for the five clinical strains exhibiting the lowest attachment capacities (LAC-01 to -05) versus the mean percentage for the five clinical strains with the highest attachment capacities (HAC-01 to -05). The eight values obtained for each strain were presented in box-and-whisker plots obtained via Statgraphic Plus 5.1 software. The crosses (+) indicate the mean values of the data distributions.

Besides their differences with *P. aeruginosa* PAO1, our results also revealed a high heterogeneity among the PLDs of clinical strains. Indeed, the PCA performed with the PL quantitation data showed an angle close to 180° between the PLDs’ vectors of the “LAC” and “HAC” groups, whereas each of these vectors formed an angle close to 90° with the “CF group” PLD vector (cf. *Materials and Methods* section for precision) (Figure 2). These results indicated major differences between the PLDs of these three groups of clinical strains. The PCA analysis grouped HAC-01/-04/-05, HAC-02/-03 and LAC-01/-02/-03 according to their PLD, whereas LAC-04 and LAC-05 seemed to each possess a unique PL profile. This result indicated the existence of 5 different profiles among the PLDs of the 10 clinical strains studied (Figure 2). The clustering of HAC-01/-04/-05 on the one hand and of HAC-02/-03 on the other hand is in agreement with an analysis of the variability among the HAC strains performed at the PL level using a Kruskal–Wallis test and a box-and-whisker plot. Except for PC 32:0 and PE 16:0-16:0, all *p*-values were <0.05 and the plots showed that, 21 times out of 28 analyses, that the strains HAC-01/-04/-05 or HAC-02/-03 exhibited the same data distributions (Table S1: “Clinical strains variability” sheet and Figure S1). Overall, these results show that the clinical strains sharing the same HAC phenotype are heterogeneous in their PL profiles, but that this heterogeneity is limited to two main PL profiles. Regarding the “LAC group”, the PCA distributed the clinical strains in three sub-groups: LAC-01/-02/-03, LAC-05 alone and LAC-04, which was also isolated among LAC strains and clustered with PAO1 (Figure 2). As for the “HAC group”, this result indicated that the PLDs of the LAC strains were heterogeneous but at a higher degree. Indeed, all Kruskal–Wallis *p*-values were significative and no consensual subgroups were identified from the analysis of the box-and-whisker plots (Table S1: “Clinical strains variability” sheet and Figure S1). By comparing the clinical isolates strain-by-strain, considering all data, we observed that the ratio of extreme values for each PL was always superior to 2, even superior to 10 for 9 PL, between two clinical strains with a maximum value of 42.8, except for the main PE species, PE 16:0-18:1, for which the ratio’s maximum/minimum value was 1.6. So our results showed that the PL profiles of cystic fibrosis clinical strains of *P. aeruginosa* were highly variable, even among strains sharing a particular phenotype, in our case the attachment capacity. This PLDs’ variability was much more important among clinical isolates than when comparing them to PAO1, in terms of the number of PLs concerned and the amplitude of the modifications. Despite this variability, we attempted to identify PLs with a homogeneous quantity within the “LAC” or “HAC” groups but systematically different between the two groups. To do this, the approach used to compare the PLDs of PAO1 and the “CF group” was applied. So, we identified 3 PLs, all belonging to the PE class, in which the relative quantities were statistically different between the “LAC” and “HAC” groups, with a minimal factor of two between the two groups (Figure 3). The first, PE 16:0-17:1, represented an average of 4.4% ± 2.1% and the sixth rank of the quantified PE in the “HAC group”, whereas it was 2.9 less present in the “LAC group”, with a relative amount of 1.5% ± 0.7% and the ninth rank (Table 1 and Table 2). The box-and-whisker plot showed that this result was not a constant difference between the LAC and HAC strains but was due to the HAC-01/-04/-05 cluster for which the main relative quantity of PE 16:0-17:1 was 6.1 ± 0.1% (Figure 3A). The second PL, PE 17:1-19:1, represented 2.0% ± 1.5% and took the ninth rank of the quantified PE in the “HAC group”. Its percentage in the “LAC group” was reduced by a factor of 2.3 (0.9% ± 0.9%), which brought this PL to the 11th place (Table 1 and Table 2). In this case, the box-and-whisker plot showed both the PLD heterogeneity among the clinical strains but also within the “LAC” or “HAC” groups. So this PL was selected because of a difference between LAC-01/-02/-03/-05 plus HAC-02/-03 on the one hand and HAC-01/-04/-05 plus LAC-04 on the other hand, and so it is without correlation with the AC phenotype (Figure 3B). The last PL, which may be differentially accumulated in relation with an AC phenotype, was PE 18:1-18:1. The average relative quantity of this PL was higher in the “LAC group” (2.0% ± 0.4%) than in the “HAC group” (0.9% ± 0.2%), i.e., a modification factor of 2.3, which moved PE 18:1-18:1 from the seventh to the ninth rank, respectively (Table 1 and Table 2). This time, the box-and-whisker plot showed relative heterogeneity within the “LAC” and “HAC” groups but a clear separation of all the LAC strains from all the HAC strains (Figure 3C). These observations, and the fact that there are significant differences between the data of any LAC strain vs. any HAC strain (data not shown), make PE 18:1-18:1 a potential molecular actor involved in attachment, the first step in biofilm formation, in clinical *P. aeruginosa* strains.

## 10. Repeatability of Data

**Figure 4:**
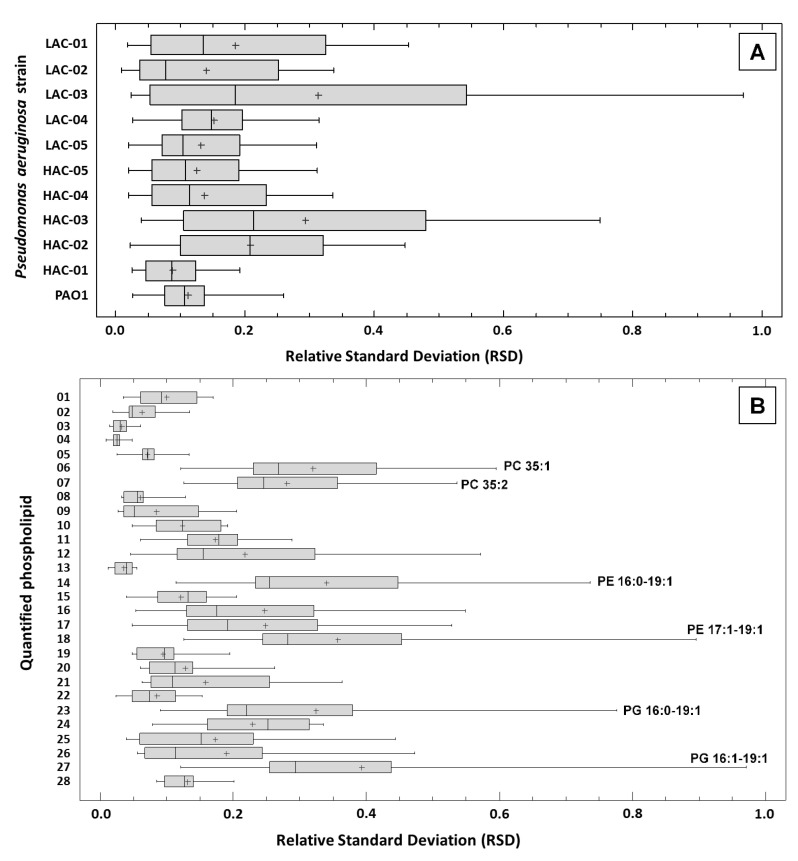
Repeatability of the phospholipids’ relative quantitation. Cells of each of the 11 *Pseudomonas aeruginosa* strains (10 clinical + PAO1) studied were harvested after 18 h of incubation on lysogeny broth (LB) agar. Each strain was grown in separate octuplates (*n* = 8) and phospholipids (PLs) were extracted, leading to the quantitation of 28 PL species as detailed in the *Materials and Methods* section. The relative standard deviation (RSD) of the relative quantifications was used as the parameter for estimating experimental variability (Table S1). The analysis of the repeatability for each strain (**A**) took into account the 28 RSDs established for each PL quantified in this study, and the analysis of the repeatability for PL molecular species (**B**) took into account the 11 RSDs established for each strain. For each analysis, the RSDs’ distributions were presented in box-and-whisker plots obtained via Statgraphic Plus 5.1 software. The crosses (+) indicate the mean of the RSD distributions. (**B**): 01: PC 32:0; 02: PC 32:1; 03: PC 34:0; 04: PC 34:1; 05: PC 34:2; 06: PC 35:1; 07: PC 35:2; 08: PE 14:0-18:1; 09: PE 15:0-18:1; 10: PE 16:0-16:0; 11: PE 16:0-16:1; 12: PE 16:0-17:1; 13: PE 16:0-18:1; 14: PE 16:0-19:1; 15: PE 16:1-18:1; 16: PE 16:1-19:1; 17: PE 17:1-18:1; 18: PE 17:1-19:1; 19: PE 18:1-18:1; 20: PG 16:0-16:0; 21: PG 16:0-17:1; 22: PG 16:0-18:1; 23: PG 16:0-19:1; 24: PG 16:1-18:1; 25: PG 16:1-19:1; 26: PG 17:1-18:1; 27: PG 17:1-19:1; 28: PG 18:0-18:1.

An important point to validate a result is its reliability. Our work is based on the realization of eight biological replicates (octuplate) per strain of *P. aeruginosa* in order to be able to use at least nonparametric statistical tests. In the continuity of the quality control of our results, we estimated the quantitation repeatability of the 28 PL molecular species analyzed in our study. The relative standard deviation (RSD) was used as an indicator of experimental repeatability. A total of 308 RSDs were calculated, i.e., one per PL per strain from the eight independent quantitation data (Table S1: “RSD” sheet). Overall, the mean value of the 308 RSDs was 17.2% ± 15.5% (min: 0.9%–max: 97.1%), whereas the median and the third quartile values were 12.6% and 23.6%, respectively. This indicated good repeatability of the data in general. Nevertheless, the highest RSDs were highlighted for some octuplates. The box-and-whisker plots allowed us to visualize the RSDs’ distributions for each *P. aeruginosa* strain, using the 28 PLs RSDs (Figure 4A), and for each PL, using the 11 strains of RSDs (Figure 4B). The highest RSDs (>50%) were found in 2 strains out of 11 and concerned 9 PLs out of 28. The highest variability in PL quantitation results was observed only for the strains LAC-03 and HAC-03. In LAC-03, the RSDs for PE 17:1-19:1, PG 16:0-19:1 and PG 17:1-19:1 were >75%, whereas those of PC 35:1, PC 35:2, PE 16:0-17:1, PE 16:0-19:1, PE 16:1-19:1 and PE 17:1-18:1 ranged from 50% to 75%. In HAC, no RSD exceeded 75% and those of PC 35:2, PE 16:0-19:1, PE 17:1-19:1, PG 16:0-19:1 and PG 17:1-19:1 ranged from 50% to 75%. Despite those high values, the mean RSD of LAC-03 and HAC-03 were 31.3% ± 28.8% (median: 18.5%) and 29.4% ± 21.1% (median: 21.4%), respectively. Concerning the other nine strains, the mean RSDs ranged from 8.8% ± 4.3% to 20.9% ± 12.9%. This analysis of the RSDs indicated good repeatability of the biological replicates and moderate variation in the data, as more than 75% of the RSDs were inferior to 25%. Of particular interest was the fact that RSDs of PE 18:1-18:1 through all strains ranged from 4.6% to 19.5% (mean: 9.4% ± 4.2%); that validates all quantitation and results for this PL.

## 11. Discussion

The formation of a biofilm is a sequential process including four major steps: the attachment, microcolony formation, maturation and dispersion phases [21]. The attachment phase encompasses all the events permitting bacteria to come onto contact with a surface and switch from a planktonic to a sessile state, resulting in its irreversible attachment onto this surface. We determined the attachment capacities (AC) of *P. aeruginosa* PAO1 and of clinical strains isolated from the sputa of cystic fibrosis (CF) patients. First, in our experimental conditions, we showed that the AC of *P. aeruginosa* PAO1 could be qualified as “average”, as just under one in two cells of a bacterial suspension of this collection strain adhered to the glass wool (GW) fibers within 20 min of incubation (i.e., AC = 41%). Heterogeneity within a bacterial suspension, even made from a single colony from an agar plate, has been described for a long time, questioning the simplistic view of a homogeneous culture of genetically identical bacteria commonly found in microbiology [22]. Similarly, the heterogeneity of AC was recently described within a clonal suspension of *Escherichia coli* containing cells that did not adhere to borosilicate glass, and adhering cells themselves exhibit a phenotypic heterogeneity in their adhesion strength [23]. Regarding the 159 clinical strains tested, the AC values were highly variable, ranging from ≈1% to ≈91%, but not uniformly distributed, as 147 strains (92%) had an AC less than 50%. Such variability is described in the late phases of biofilm formation. Thus, Murray et al. [24] showed that clinical isolates of *P. aeruginosa* have a capacity to form biofilms ranging from 14% to 1000%, compared to that of PAO1 with a median value close to 96%, whereas Milivojevic et al. [25] showed that a majority of *P. aeruginosa* isolated from humans or animals formed fewer biofilms than the PAO1 reference strain. Such quantitative differences in biofilm formation between clinical and collection strains have been also described in other organisms like *Acinetobacter baumannii*, *Staphylococcus epidermidis* and *Listeria monocytogenes* [26,27,28]. This dichotomy could be explained by the domestication of strains, a process in which a bacterial strain accumulates mutations over time compared to its original “ancestral” environmental strain because of the growth conditions used in laboratories [29]. For instance, Barreto et al. [30] showed that a mutation in *degU,* a gene coding for a transcriptional regulator, occurs after only 16 days of daily subculture in a laboratory in an environmental *Bacillus subtilis* strain, impairing its formation of a robust biofilm architecture like its ancestor formed. Beyond the difference between the ACs of the PAO1 reference strain and the clinical isolates, our results also show variability among the latter, which nevertheless all have the same degree of domestication. Such disparity has been described by Deligianni et al. [31] who showed that CF *P. aeruginosa* isolates exhibited diversity in their biofilm quantities and architectures without a systematic link to genotype diversity. Our results showed for the first time that the AC of a large majority of clinical isolates of *P. aeruginosa* is modest compared to the AC of the collection strain PAO1. This result does not presume the ability of the same strains to form mature biofilms as no direct relationship exists between the amount of biofilm formed and the initial adhesion extent [32].

In the context of our analysis of the attachment phase, we selected 10 *P. aeruginosa* strains with extreme and opposite ACs, forming the so-called “CF group” of strains. This group is composed of two sub-groups: The “Low Attachment Capacity” group (LAC) groups five clinical isolates with ACs less than 5%, and the “High Attachment Capacity” group (HAC) is composed of five strains with ACs greater than 85%. To identify potential molecular actors involved in the attachment phase, we experimentally determined the phospholipid (PL) profiles, named phospholipidome (PLD), of the 10 clinical strains as well as the PL profile of the reference strain PAO1; then we generated in silico PLDs of the “CF”, “LAC” and “HAC” groups and compared the PAO1 to “CF group” PLDs, as well as “LAC” and “HAC” group PLDs. Phospholipids are major structural compounds of bacterial membranes and play an important functional role by controlling exchanges between the intra- and extracellular compartments and regulating the spatio-temporal position of membrane proteins and protein complexes that play an essential role in many cellular functions [33]. Moreover, it has been shown that alterations in a cell PL profile lead to the modification of several phenotypes like bacterial fitness, antibiotic resistance or the ability to form biofilms [34,35,36]. In our work, the relative quantities of 28 PL species were determined for PAO1 and the 10 clinical strains. All PLs belonged to phosphatidylethanolamines (PE), phosphatidylglycerols (PG), and phosphatidylcholines (PC), the main PLs composing cellular membrane vesicles and outer membranes in *P. aeruginosa* [37]. Phosphatidylserines, phosphatidic acids and phosphatidylinositols were not detected, presumably because of their low content in bacterial membranes.

Our results showed a clear statistical difference in the relative quantities of numerous PLs between *P. aeruginosa* PAO1 and the clinical isolates. Indeed, more than 80% (226/280) of the Mann–Whitney tests indicated a statistical differences between the collection and the clinical strains for any of the 28 PLs quantified, and no PL presented a constant *p*-value > 0.05. Having differences between the PLDs of PAO1 and clinical strains is not surprising. It has been shown that *P. aeruginosa* CF strains exhibit phenotypic and genotypic differences because of adaptation processes leading to the emergence of strains often characterized by alginate overproduction, impaired motility, a quorum-sensing deficiency, rough lipopolysaccharides, diminished secretion of protease and hypermutation [38,39]. Recently, Deschamps et al. [40] demonstrated that a *P. aeruginosa* collection strain growing in a CF sputum-like medium had a distinct PLD compared to its counterpart grown in a complex medium classically used in laboratories, showing that environmental nutritional conditions in lungs may by itself induce modification of the PLD. The most surprising part of our result is the extent of differences. By comparing the “CF group” to PAO1, our results showed that most PLDs of the clinical isolates are enriched in PC 32:0, PE 16:0-16:0, PE 16:0-16:1 and PG 16:0-16:0 but also that these strains contained less of certain PLs than PAO1: PC 35:1, PC 35:2, PE 16:0-19:1, PE 18:1-18:1 and PG 18:0-18:1. Our results also highlighted that the relative abundance of a PL is often diminished in a clinical strain compared to PAO1 but often by a modest factor. Thus we showed the PLD variability between a collection and several clinical strains of *P. aeruginosa*, independent of the growth conditions. Recently, Deschamps et al. [40] warned researchers to pay attention to the growth conditions used in lipidomic studies. Our results reinforce this warning by showing that the origin and history of the strain used in lipidomic studies may also be important to consider.

The variability of the PL profiles among the clinical isolates was also revealed by our study. The PCA analysis grouped the strains HAC-01/-04/-05, HAC-02/-03 and LAC-01/-02/-03 according to their PLD, whereas LAC-04 and LAC-05 seemed to each possess a unique PL profile. This result indicated the existence of 5 different profiles among the PLDs of the 10 clinical strains. Nevertheless, important variations punctually distinguished strains sharing a “globally common” profile. For instance, among the strains, in the cluster LAC-01/-02/-03, the PE 15:0-18:1 relative quantity varied by a factor >6 between LAC-01 and LAC-02/-03. It should be noticed that this variation is not linked to a problem with the repeatability of data, as the RSDs in this case are all less than 9%. This observation reinforces the necessary vigilance with regards to the choice of a strain for lipidomic analysis. If the variation of the PL composition according to the bacterial species or the environmental conditions, and the effect of this variation on phenotypes such as virulence [41], are accessible in the literature, few publications described differences in *P. aeruginosa* PL profiles in mature biofilms [42,43] and, to our knowledge, only one concerned the attachment phase [16]. All these studies were based on a comparison of planktonic versus sessile cells of a collection strain. Here we tested an alternative strategy to identify potential molecular actors involved in the attachment phase, in the aim to fight against biofilms. This strategy is based on the comparison of two pools of clinical strains sharing a common phenotype regarding their AC within a pool but presumed genetically different between and within the pools [44,45]. Thus, in an ideal situation, a PL having a constant relative quantity within strains of the same pool (LAC or HAC) but systematically differentiating the strains of different pools (LAC vs. HAC) might be a molecular actor linked to the attachment phenotype and therefore, a potential molecular target. If existing, such target is constitutively expressed or repressed by strains of the same pool, and its expression/repression may be causal, and not a consequence, of the studied phenotype. In our case, by comparing the strains of the “LAC group” to those of the “HAC group” with stringent parameters (statistical test + factor ≥ 2), three PEs were identified as potentially interesting PLs to investigate in our study of attachment. The two first, PE 16:0-17:1 and PE 17:1-19:1, have a higher relative quantity in the “HAC group” compared to the “LAC group”, but a deeper analysis revealed heterogeneity within one or both pools, respectively, excluding the possibility of establishing a potential link between the attachment phenotype and the quantitative variability of these PLs. The last PL, PE 18:1-18:1, was more interesting. Indeed, the mean relative quantity of this PL was lower in strains of the “HAC group” compared to those of the “LAC group” and, despite variability within each pool, we observed a clear discrimination of strains according to their AC. This result is in accordance with our previous analysis of the PLD of PAO1 immobilized on GW fibers showing that the relative amount of this PL was lower in the attached PAO1 cells compared to the unattached cells, as of five minutes of incubation [16]. PE 18:1-18:1, dioleoylphosphatidylethanolamine (DOPE), belongs to the PE class. PEs are zwitterionic molecules at physiological pH and are the most abundant PL class in Gram-negative bacteria. PEs are asymmetrically distributed in bacterial membranes, more abundant in the inner leaflet of both cytoplasmic and outer membranes of *E. coli* and, because of their structural and thermodynamic properties, are concentrated in the concave part of the membranes. This distribution of PE in membranes is dynamic, probably metabolically regulated, leading to the modulation of the cell shape [46]. Beyond its possible involvement in determining the shape of the cell, the specific functions of DOPE remain unclear. DOPE regulates the internalization of the glucose receptor GLUT4 in eukaryotic cells [47], and most of the information available in literature describe its role as a helper colipid in drug delivery systems as a lipoplex, a non-viral vector used to transduce eukaryotic cells [48], or liposomes, used to fight against bacterial biofilms [49]. In such structures, it has been showed that the addition of DOPE resulted in diminished water permeability by increasing the lipid packing density [50]. So the biological and/or structural impact of the reduced relative amount of DOPE in the clinical isolates exhibiting highest ACs may be linked to a modification of the membranes’ properties in these *P. aeruginosa* strains compared to those containing more DOPE.

In conclusion, our study revealed huge variability in attachment capacity, the first step in biofilm formation, of *P. aeruginosa* strains harvested from the sputa of cystic fibrosis patients, as well as a general reduced capacity to colonize glass wool fibers in cystic fibrosis clinical isolates compared to the PAO1 collection strain. We also report a high difference between the phospholipid profile of the *P. aeruginosa* PAO1 strain and those of 10 clinical isolates and the high versatility of these phospholipidomes between the clinical isolates even within strains sharing similar attachment capacities. This variability concerned each of the 28 phospholipids species quantified in this work and was more marked between two clinical strains than between a clinical strain and PAO1. By comparing the phospholipid profiles of clinical strains clustered according their attachment capacities, we identified a phospholipid, PE 18:1-18:1, which may be linked to this phenotype and so represents a potential target in the fight against biofilms. Our results illustrate the importance of the choice of strain for lipidomic analyses, the interest in investigating several strains in parallel and the need to more accurately understand the biological rules of phospholipids in order to decipher their implications in biofilm formation, in particular during the attachment phase.

## Figures and Tables

**Table 1:** Phospholipids quantitation of *Pseudomonas aeruginosa* collection and clinical strains. The quantitation of a phospholipid (PL) was expressed as its relative quantity among all phospholipids quantified of the same class (%Mol). For each PL quantified in a cystic fibrosis (CF) clinical or collection strain of *P. aeruginosa*, the indicated value is the mean of eight biological replicates ± standard deviation. Additionally, the rank of a PL is indicated between brackets. This value indicates the ranking of a PL according its relative abundance within a PL class. For instance, the rank of PC 34:1 is 1 in 7 because its relative quantity is the greatest of the seven quantified phosphatidylcholines. ^(1)^: The attachment capacity of a strain is indicated in square brackets.

	Phospholipidomes Experimentally Determined [Mean %mol. ± SD (Rank)]											In Silico Phospholipidomes [Mean %mol ± SD (Rank)]		
	«Low Attachment Capacity» (LAC) CF Strains					«High Attachment Capacity» (HAC) CF Strains					Collection			
	01 [1%] ^(1)^	02 [2%]	03 [2%]	04 [2%]	05 [3%]	05 [85%]	04 [87%]	03 [89%]	02 [90%]	01 [91%]	PAO1 [41%]	LAC Group	HAC Group	CF Group
PC 32:0	11.2 ± 1.0 (3)	10.3 ± 0.4 (3)	10.6 ± 0.6 (3)	8.5 ± 1.4 (4)	7.9 ± 0.6 (3)	12.2 ± 1.2 (4)	12.6 ± 0.8 (2)	13.0 ± 2.0 (2)	11.9 ± 1.7 (2)	12.6 ± 1.6 (3)	4.5 ± 0.4 (6)	9.7 ± 1.6 (3)	12.5 ± 1.6 (2)	11.1 ± 2.1 (3)
PC 32:1	7.2 ± 0.3 (4)	8.5 ± 0.2 (4)	8.7 ± 0.2 (4)	6.6 ± 0.4 (5)	6.8 ± 0.6 (4)	12.3 ± 0.8 (3)	11.8 ± 0.5 (4)	11.3 ± 1.5 (4)	9.6 ± 1.2 (4)	12.3 ± 0.6 (4)	5.0 ± 0.2 (5)	7.6 ± 1.0 (4)	11.5 ± 1.4 (4)	9.5 ± 2.3 (4)
PC 34:0	7.1 ± 0.2 (5)	6.6 ± 0.1 (5)	6.4 ± 0.2 (5)	6.4 ± 0.3 (6)	6.6 ± 0.2 (5)	5.4 ± 0.1 (6)	5.3 ± 0.1 (6)	6.3 ± 0.4 (5)	6.6 ± 0.3 (5)	5.5 ± 0.2 (6)	6.7 ± 0.2 (4)	6.6 ± 0.3 (5)	5.8 ± 0.6 (5)	6.2 ± 0.6 (5)
PC 34:1	57.2 ± 1.1 (1)	57.3 ± 0.5 (1)	56.5 ± 1.4 (1)	53.3 ± 1.5 (1)	57.9 ± 1.2 (1)	47.7 ± 1.4 (1)	46.4 ± 1.0 (1)	55.2 ± 2.6 (1)	57.1 ± 2.2 (1)	47.3 ± 1.2 (1)	55.3 ± 1.4 (1)	56.5 ± 2.0 (1)	50.7 ± 4.8 (1)	53.6 ± 4.7 (1)
PC 34:2	12.8 ± 0.3 (2)	15.2 ± 0.6 (2)	14.4 ± 1.2 (2)	13.2 ± 1.8 (2)	15.7 ± 1.4 (2)	13.4 ± 1.0 (2)	12.5 ± 0.9 (3)	11.9 ± 0.8 (3)	10.9 ± 0.7 (3)	13.0 ± 0.8 (2)	11.3 ± 0.8 (3)	14.2 ± 1.6 (2)	12.3 ± 1.2 (3)	13.3 ± 1.7 (2)
PC 35:1	3.1 ± 1.1 (6)	1.4 ± 0.3 (6)	2.3 ± 1.3 (6)	8.7 ± 2.0 (3)	3.5 ± 1.0 (6)	6.0 ± 1.4 (5)	7.5 ± 2.0 (5)	01.7 ± 1.0 (6)	2.9 ± 1.2 (6)	6.1 ± 1.0 (5)	12.9 ± 1.6 (2)	3.8 ± 2.9 (6)	4.9 ± 2.6 (6)	4.3 ± 2.8 (6)
PC 35:2	1.4 ± 0.5 (7)	0.7 ± 0.1 (7)	1.0 ± 0.6 (7)	3.2 ± 0.9 (7)	1.6 ± 0.4 (7)	3.1 ± 0.7 (7)	3.9 ± 0.9 (7)	0.7 ± 0.3 (7)	1.0 ± 0.4 (7)	3.1 ± 0.4 (7)	4.4 ± 0.6 (7)	1.6 ± 1.0 (7)	2.4 ± 1.4 (7)	2.0 ± 1.3 (7)
PE 14:0-18:1	1.4 ± 0.1 (9)	1.3 ± 0.0 (7)	1.4 ± 0.0 (9)	1.5 ± 0.1 (11)	1.5 ± 0.1 (10)	1.5 ± 0.1 (10)	1.4 ± 0.1 (10)	1.2 ± 0.1 (7)	1.2 ± 0.1 (8)	1.5 ± 0.1 (10)	1.5 ± 0.2 (11)	1.4 ± 0.1 (10)	1.4 ± 0.2 (10)	1.4 ± 0.1 (10)
PE 15:0-18:1	0.1 ± 0.0 (12)	0.6 ± 0.0 (11)	0.6 ± 0.0 (11)	1.0 ± 0.2 (12)	0.6 ± 0.0 (12)	1.2 ± 0.0 (11)	1.2 ± 0.0 (11)	0.4 ± 0.1 (11)	0.5 ± 0.1 (11)	1.2 ± 0.0 (11)	0.7 ± 0.1 (12)	0.6 ± 0.3 (12)	0.9 ± 0.4 (11)	0.7 ± 0.4 (12)
PE 16:0-16:0	8.2 ± 1.0 (3)	7.4 ± 0.4 (3)	7.4 ± 0.4 (3)	7.2 ± 0.9 (4)	6.0 ± 0.6 (4)	8.4 ± 1.2 (4)	9.8 ± 1.1 (4)	10.0 ± 1.8 (3)	8.5 ± 1.6 (3)	8.6 ± 1.6 (4)	3.8 ± 0.3 (6)	7.2 ± 1.0 (3)	9.1 ± 1.7 (3)	8.1 ± 1.7 (3)
PE 16:0-16:1	4.9 ± 0.4 (5)	5.8 ± 0.4 (4)	5.3 ± 0.9 (4)	3.2 ± 0.6 (6)	3.2 ± 0.6 (5)	6.9 ± 1.3 (5)	5.4 ± 1.6 (6)	8.8 ± 1.8 (4)	7.2 ± 1.7 (4)	6.4 ± 0.8 (5)	2.5 ± 0.4 (8)	4.5 ± 1.2 (5)	6.9 ± 1.9 (5)	5.7 ± 2.0 (5)
PE 16:0-17:1	1.3 ± 0.4 (10)	0.9 ± 0.2 (9)	1.6 ± 0.9 (8)	2.3 ± 0.3 (8)	1.6 ± 0.2 (9)	6.0 ± 0.6 (6)	6.2 ± 0.7 (5)	1.7 ± 0.6 (6)	2.1 ± 0.5 (6)	6.1 ± 0.3 (6)	2.3 ± 0.4 (10)	1.5 ± 0.7 (9)	4.4 ± 2.1 (6)	3.0 ± 2.1 (6)
PE 16:0-18:1	56.7 ± 2.3 (1)	58.9 ± 0.7 (1)	56.8 ± 2.7 (1)	48.5 ± 2.5 (1)	54.1 ± 1.2 (1)	39.2 ± 1.7 (1)	38.0 ± 1.3 (1)	55.6 ± 2.2 (1)	56.5 ± 1.3 (1)	38.8 ± 1.0 (1)	43.2 ± 2.4 (1)	55.0 ± 4.1 (1)	45.6 ± 8.7 (1)	50.3 ± 8.2 (1)
PE 16:0-19:1	5.5 ± 2.0 (4)	2.3 ± 0.8 (5)	4.7 ± 3.5 (5)	13.9 ± 3.3 (2)	8.4 ± 2.0 (3)	11 ± 2.6 (3)	13.2 ± 3.4 (2)	2.8 ± 1.8 (5)	4.8 ± 2.2 (5)	11.7 ± 1.6 (3)	16.0 ± 1.8 (2)	7.0 ± 4.7 (4)	8.7 ± 4.8 (4)	7.8 ± 4.8 (4)
PE 16:1-18:1	16.1 ± 0.8 (2)	18.5 ± 0.7 (2)	16.9 ± 2.6 (2)	12.2 ± 2.0 (3)	15.8 ± 1.7 (2)	14.3 ± 2.3 (2)	12 ± 2.5 (3)	16.3 ± 1.4 (2)	14.7 ± 1.9 (2)	13.8 ± 1.3 (2)	13.4 ± 1.9 (3)	15.9 ± 2.7 (2)	14.2 ± 2.4 (2)	15.1 ± 2.7 (2)
PE 16:1-19:1	1.6 ± 0.5 (7)	0.8 ± 0.2 (10)	1.4 ± 0.7 (9)	2.3 ± 0.3 (8)	2.4 ± 0.4 (7)	3.2 ± 0.4 (8)	3.3 ± 0.5 (9)	0.7 ± 0.4 (10)	1.2 ± 0.4 (8)	3.3 ± 0.2 (8)	3.8 ± 0.4 (6)	1.7 ± 0.8 (8)	2.4 ± 1.2 (8)	2.0 ± 1.1 (8)
PE 17:1-18:1	2.0 ± 0.7 (6)	1.2 ± 0.3 (8)	1.9 ± 1.0 (6)	3.3 ± 0.5 (5)	3.2 ± 0.6 (5)	4.8 ± 0.6 (7)	5.1 ± 0.8 (7)	1.0 ± 0.5 (9)	1.6 ± 0.5 (7)	4.9 ± 0.2 (7)	5.4 ± 0.6 (4)	2.3 ± 1.1 (6)	3.5 ± 1.9 (7)	2.9 ± 1.6 (7)
PE 17:1-19:1	0.6 ± 0.3 (11)	0.2 ± 0.0 (12)	0.4 ± 0.3 (12)	2.5 ± 0.7 (7)	0.8 ± 0.3 (11)	2.8 ± 0.7 (9)	3.6 ± 1.0 (8)	0.3 ± 0.2 (12)	0.5 ± 0.2 (11)	2.9 ± 0.4 (9)	2.5 ± 0.4 (8)	0.9 ± 0.9 (11)	2.0 ± 1.5 (9)	1.5 ± 1.4 (9)
PE 18:1-18:1	1.6 ± 0.1 (7)	2.1 ± 0.1 (6)	1.7 ± 0.3 (7)	2.1 ± 0.3 (10)	2.4 ± 0.3 (7)	0.7 ± 0.0 (12)	0.7 ± 0.0 (12)	1.1 ± 0.1 (8)	1.1 ± 0.1 (10)	0.8 ± 0.1 (12)	4.8 ± 0.3 (5)	2.0 ± 0.4 (7)	0.9 ± 0.2 (11)	1.4 ± 0.6 (10)
PG 16:0-16:0	7.5 ± 1.0 (4)	6.3 ± 0.4 (3)	6.5 ± 0.4 (4)	7.1 ± 0.8 (4)	5.4 ± 0.4 (5)	8.2 ± 0.8 (4)	7.7 ± 1.0 (4)	6.9 ± 1.8 (3)	5.5 ± 1.3 (4)	7.9 ± 1.0 (5)	3.4 ± 0.4 (8)	6.6 ± 1.0 (4)	7.2 ± 1.6 (5)	6.9 ± 1.3 (4)
PG 16:0-17:1	3.6 ± 0.9 (6)	2.2 ± 0.4 (6)	3.2 ± 1.2 (6)	4.7 ± 0.4 (7)	3.4 ± 0.3 (6)	8.2 ± 0.5 (4)	7.3 ± 0.5 (5)	3.8 ± 1.1 (5)	4.7 ± 0.5 (5)	7.7 ± 0.8 (6)	3.7 ± 0.4 (7)	3.4 ± 1.1 (6)	6.3 ± 1.9 (6)	4.9 ± 2.1 (6)
PG 16:0-18:1	53.1 ± 3.9 (1)	63.1 ± 1.5 (1)	59.4 ± 6.3 (1)	35.9 ± 5.5 (1)	49.5 ± 3.4 (1)	33.6 ± 3.8 (1)	30.8 ± 4.2 (1)	58.8 ± 2.4 (1)	56.0 ± 2.7 (1)	34.1 ± 2.2 (1)	41.6 ± 4.5 (1)	52.2 ± 10.4 (1)	42.7 ± 12.5 (1)	47.4 ± 12.5 (1)
PG 16:0-19:1	10.9 ± 3.9 (3)	4.1 ± 1.3 (4)	8.9 ± 6.9 (3)	28.3 ± 5.9 (2)	20.4 ± 3.9 (2)	23.1 ± 4.4 (2)	27.7 ± 6.1 (2)	6.6 ± 4.3 (4)	11.7 ± 4.4 (3)	23.3 ± 2.1 (2)	26.4 ± 4.5 (2)	14.5 ± 9.9 (2)	18.5 ± 9.1 (2)	16.5 ± 9.7 (2)
PG 16:1-18:1	15.3 ± 2.5 (2)	19.7 ± 1.7 (2)	15.2 ± 4.9 (2)	8.7 ± 2.7 (3)	9.5 ± 1.6 (3)	8 ± 2.5 (6)	7.3 ± 2.4 (5)	18.5 ± 4.1 (2)	13.7 ± 3.4 (2)	8.1 ± 0.6 (4)	7.1 ± 1.8 (4)	13.7 ± 5.0 (3)	11.1 ± 5.2 (3)	12.4 ± 5.3 (3)
PG 16:1-19:1	3.0 ± 0.6 (7)	1.5 ± 0.3 (7)	2.1 ± 0.6 (7)	2.8 ± 0.4 (8)	3.1 ± 0.2 (7)	4.1 ± 0.2 (8)	3.5 ± 0.3 (8)	1.6 ± 0.7 (7)	2.5 ± 0.6 (7)	4.0 ± 0.3 (8)	3.8 ± 0.2 (6)	2.5 ± 0.8 (7)	3.2 ± 1.1 (8)	2.8 ± 1.0 (7)
PG 17:1-18:1	5.1 ± 1.2 (5)	2.5 ± 0.6 (5)	3.7 ± 1.5 (5)	6.4 ± 0.7 (5)	6.5 ± 0.5 (4)	9.8 ± 0.7 (3)	9.2 ± 0.5 (3)	3.0 ± 1.4 (6)	4.7 ± 1.1 (5)	9.8 ± 0.6 (3)	9.3 ± 0.9 (3)	4.8 ± 1.8 (5)	7.3 ± 3.0 (4)	6.1 ± 2.8 (5)
PG 17:1-19:1	1.2 ± 0.5 (8)	0.3 ± 0.1 (8)	0.7 ± 0.7 (8)	5.9 ± 1.6 (6)	1.9 ± 0.6 (8)	4.9 ± 1.3 (7)	6.3 ± 1.7 (7)	0.6 ± 0.4 (8)	1.0 ± 0.4 (8)	4.9 ± 0.6 (7)	4.2 ± 0.8 (5)	2.0 ± 2.2 (8)	3.5 ± 2.5 (7)	2.8 ± 2.5 (7)
PG 18:0-18:1	0.2 ± 0.0 (9)	0.2 ± 0.0 (9)	0.2 ± 0.0 (9)	0.3 ± 0.0 (9)	0.3 ± 0.1 (9)	0.1 ± 0 (9)	0.1 ± 0.0 (9)	0.2 ± 0.0 (9)	0.3 ± 0 (9)	0.1 ± 0.0 (9)	0.5 ± 0.1 (9)	0.2 ± 0.1 (9)	0.2 ± 0.1 (9)	0.2 ± 0.1 (9)

**Table 2:** Quantitative variations of phospholipids between *Pseudomonas aeruginosa* strains clustered according to their origin or attachment capacities. Ten clinical isolates were isolated from cystic fibrosis patients and clustered in the so-call “CF group”. This group is composed of two subgroups: The “LAC group” clustering the five clinical strains with the lowest attachment capacities (ACs) and the “HAC group” composed of the five strains with the highest ACs. The phospholipid (PL) profiles of these 10 clinical strains as well as the PL profile of the collection strain PAO1 were determined experimentally by liquid chromatography/tandem mass spectrometry. From these data, in silico phospholipidomes were established for the “CF”, “LAC” and “HAC” groups by averaging for each PL the experimental values of all the strains included in a group. The ratios presented here were calculated from the mean data presented in Table 1 and *p*-values (Mann–Whitney test) were determined from the data obtained through eight biological replicates/strain (Table S1: PL data sheet).

	CF Group vs. PAO1 (CF/PAO1)		LAC Group vs. PAO1 (LAC/PAO1)		HAC Group vs. PAO1 (HAC/PAO1)		LAC vs. HAC Groups (LAC/HAC)	
	Ratio	*p*-Value	Ratio	*p*-Value	Ratio	*p*-Value	Ratio	*p*-Value
PC 32:0	2.5	3.5 × 10^−6^	2.2	1.0 × 10^−5^	2.8	1.0 × 10^−5^	0.8	5.4 × 10^−10^
PC 32:1	1.9	3.5 × 10^−6^	1.5	1.0 × 10^−5^	2.3	1.0 × 10^−5^	0.7	2.5 × 10^−13^
PC 34:0	0.9	3.1 × 10^−2^	1.0	3.9 × 10^−1^	0.9	1.2 × 10^−3^	1.1	1.7 × 10^−7^
PC 34:1	1.0	8.6 × 10^−1^	1.0	6.8 × 10^−2^	0.9	3.0 × 10^−2^	1.1	4.3 × 10^−6^
PC 34:2	1.2	5.2 × 10^−4^	1.3	2.8 × 10^−5^	1.1	1.6 × 10^−2^	1.2	6.9 × 10^−7^
PC 35:1	0.3	3.8 × 10^−6^	0.3	1.2 × 10^−5^	0.4	1.0 × 10^−5^	0.8	4.5 × 10^−2^
PC 35:2	0.5	3.4 × 10^−5^	0.4	2.5 × 10^−5^	>0.5	2.5 × 10^−4^	0.7	3.9 × 10^−2^
PE 14:0-18:1	0.9	1.2 × 10^−1^	0.9	2.3 × 10^−1^	0.9	7.9 × 10^−2^	1.0	9.7 × 10^−1^
PE 15:0-18:1	1.0	3.5 × 10^−1^	0.8	7.8 × 10^−3^	1.2	3.8 × 10^−1^	0.7	6.6 × 10^−3^
PE 16:0-16:0	2.2	3.5 × 10^−6^	1.9	1.0 × 10^−5^	2.4	1.0 × 10^−5^	0.8	3.4 × 10^−7^
PE 16:0-16:1	2.3	1.2 × 10^−5^	1.8	9.6 × 10^−5^	2.8	1.0 × 10^−5^	0.6	1.1 × 10^−8^
PE 16:0-17:1	1.3	6.1 × 10^−1^	0.7	2.3 × 10^−3^	1.9	3.9 × 10^−2^	0.4	1.4 × 10^−8^
PE 16:0-18:1	1.2	5.1 × 10^−2^	1.3	1.5 × 10^−5^	1.1	5.5 × 10^−1^	1.2	4.1 × 10^−6^
PE 16:0-19:1	0.5	5.6 × 10^−5^	0.4	1.4 × 10^−4^	>0.5	1.1 × 10^−4^	0.8	9.6 × 10^−2^
PE 16:1-18:1	1.1	8.0 × 10^−2^	1.2	1.7 × 10^−2^	1.1	3.5 × 10^−1^	1.1	4.7 × 10^−3^
PE 16:1-19:1	>0.5	1.8 × 10^−5^	0.4	1.3 × 10^−5^	0.6	1.4 × 10^−4^	0.7	8.1 × 10^−3^
PE 17:1-18:1	>0.5	7.6 × 10^−5^	0.4	1.2× 10^−5^	0.6	1.7 × 10^−3^	0.7	6.0 × 10^−3^
PE 17:1-19:1	0.6	4.7 × 10^−2^	0.4	3.8 × 10^−4^	0.8	8.2 × 10^−1^	0.4	8.3 × 10^−4^
PE 18:1-18:1	0.3	3.5 × 10^−6^	0.4	1.0 × 10^−5^	0.2	1.0 × 10^−5^	2.3	3.5 × 10^−14^
PG 16:0-16:0	2.0	3.5 × 10^−6^	1.9	1.0 × 10^−5^	2.1	1.0 × 10^−5^	0.9	1.3 × 10^−2^
PG 16:0-17:1	1.3	2.1 × 10^−1^	0.9	3.1 × 10^−1^	1.7	6.3 × 10^−4^	>0.5	6.5 × 10^−9^
PG 16:0-18:1	1.1	2.6 × 10^−1^	1.3	6.4 × 10^−3^	1.0	5.7 × 10^−1^	1.2	1.0 × 10^−3^
PG 16:0-19:1	0.6	5.4 × 10^−3^	>0.5	3.2 × 10^−3^	0.7	1.9 × 10^−2^	0.8	6.0 × 10^−2^
PG 16:1-18:1	1.8	3.9 × 10^−3^	1.9	1.2 × 10^−3^	1.6	2.5 × 10^−2^	1.2	1.8 × 10^−2^
PG 16:1-19:1	0.7	5.0 × 10^−3^	0.7	5.1 × 10^−5^	0.8	2.0 × 10^−1^	0.8	6.8 × 10^−4^
PG 17:1-18:1	0.7	2.9 × 10^−3^	>0.5	1.0 × 10^−5^	0.8	2.1 × 10^−1^	0.7	4.6 × 10^−4^
PG 17:1-19:1	0.7	9.1 × 10^−2^	0.5	3.5 × 10^−3^	0.8	7.7 × 10^−1^	0.6	7.2 × 10^−3^
PG 18:0-18:1	0.5	3.4 × 10^−6^	>0.5	9.7 × 10^−6^	0.4	9.5 × 10^−6^	1.2	4.9 × 10^−4^

## Data Availability

Data supporting reported results can be found in Table S1. Mass spectrometry data are available under request to the authors.

## Supplementary Materials

The following are available online at https://www.mdpi.com/article/10.3390/ijms22084003/s1, Figure S1: Distributions of phospholipid quantitation data from 8 biological replicates per strain., Table S1: Experimental data.
