# Supplementary material for: Analysis of the Phospholipid Profile of the Collection Strain PAO1 and Clinical Isolates of Pseudomonas aeruginosa in Relation to Their Attachment Capacity
Source: Int J Mol Sci. 2021 Apr 13;22(8):4003. doi: 10.3390/ijms22084003 (PMC8068974; doi:10.3390/ijms22084003)
Supplement: Supplementary file 1 [file ijms-22-04003-s001.zip › FigureS1.pdf]

Figures S1: Distributions of phospholipid quantitation data from 8 biological replicates per strain. “LAC” stands for “Low Attachment Capacity” strain whereas “HAC” means “High Attachment Capacity”. Numbers after these acronyms represent the clinical strain number. Quantitation data corresponded to the relative amount of a phospholipid among all the quantified phospholipids of the same class. The distributions are presented in box-and-whisker plots obtained via Statgraphic Plus 5.1 software. One plot was performed for each phospholipid quantified in our study. The crosses (+) indicate the means of the distributions.

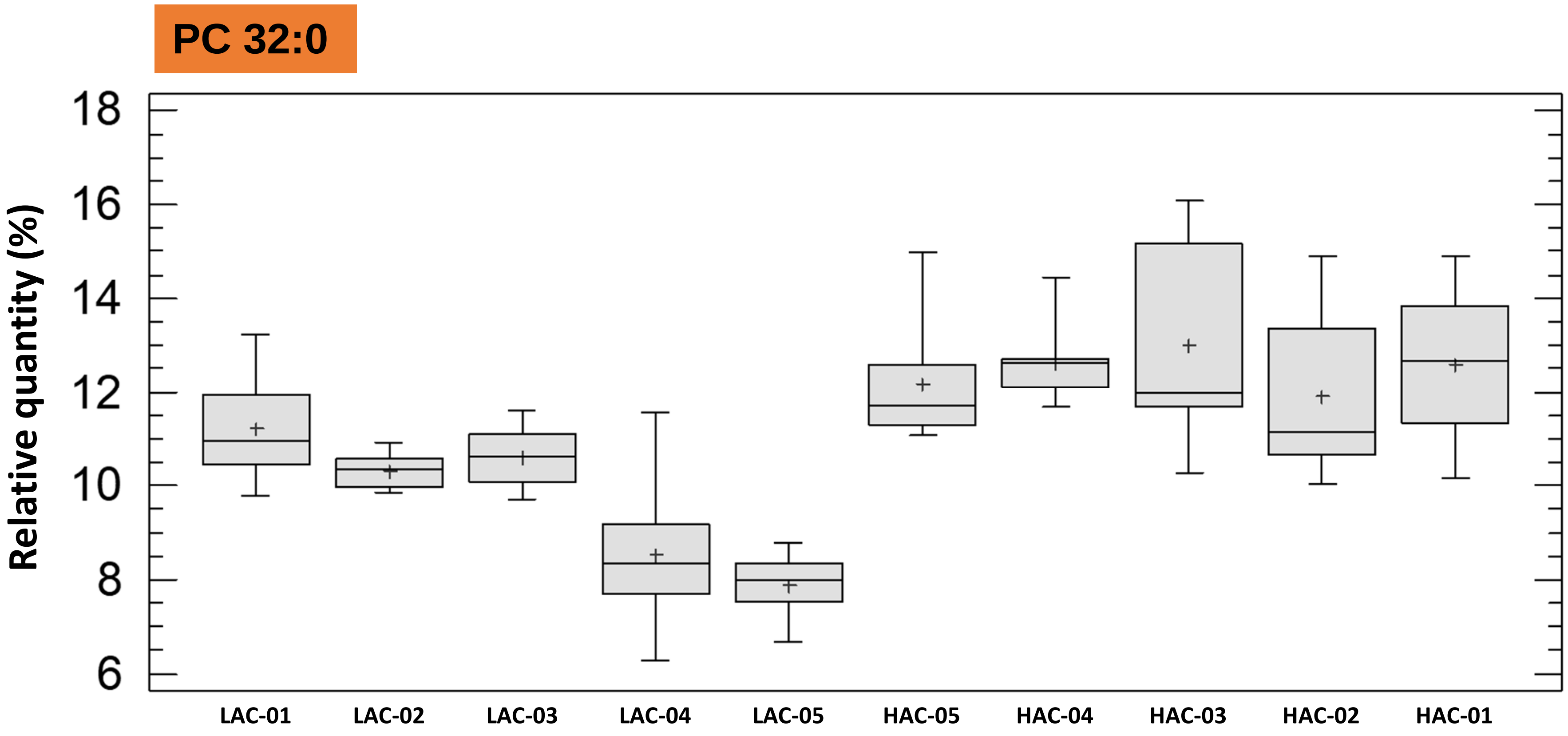

PC 32:0
Relative quantity (%)
18
16
14
12
10
8
6
LAC-01
LAC-02
LAC-03
LAC-04
LAC-05
HAC-05
HAC-04
HAC-03
HAC-02
HAC-01

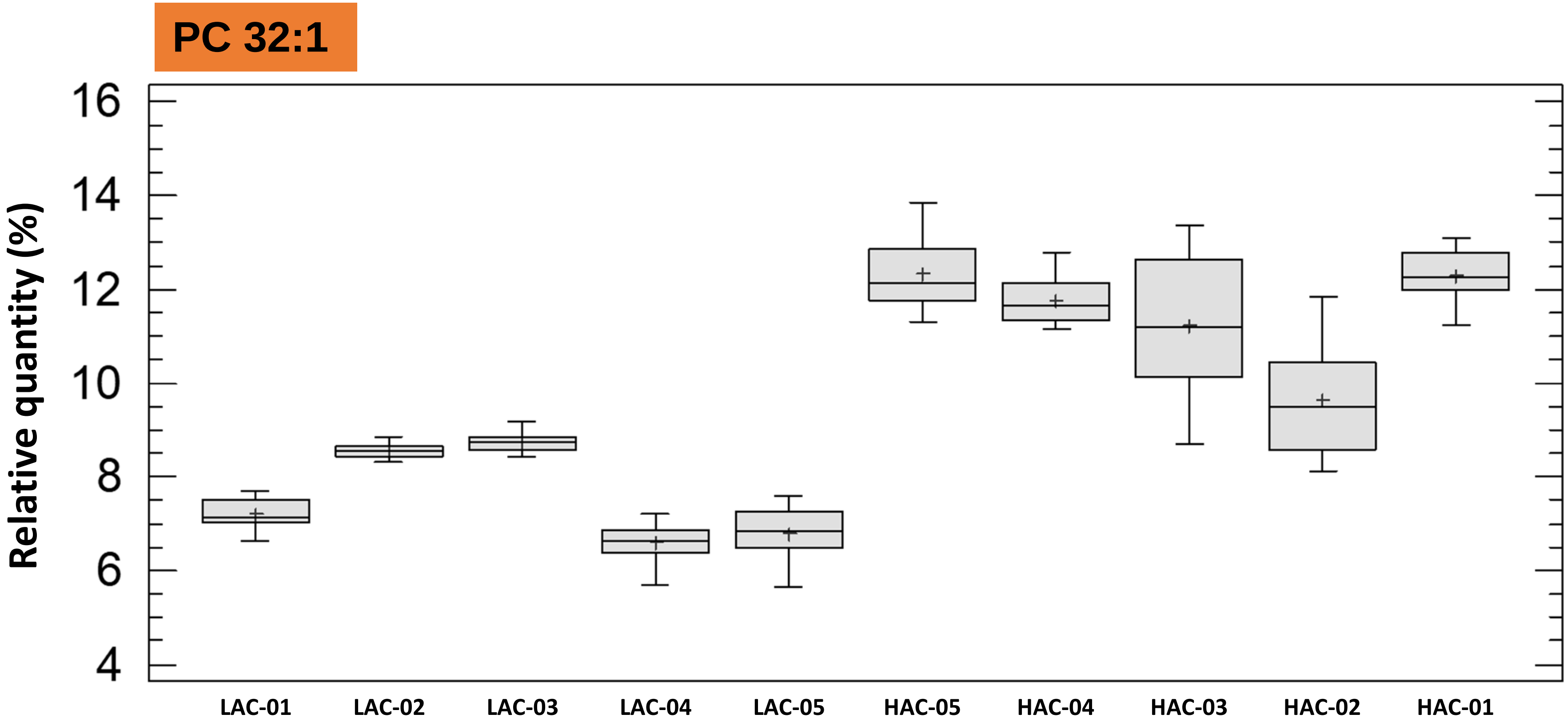
PC 32:1
Relative quantity (%)
16
14
12
10
8
6
4
LAC-01
LAC-02
LAC-03
LAC-04
LAC-05
HAC-05
HAC-04
HAC-03
HAC-02
HAC-01

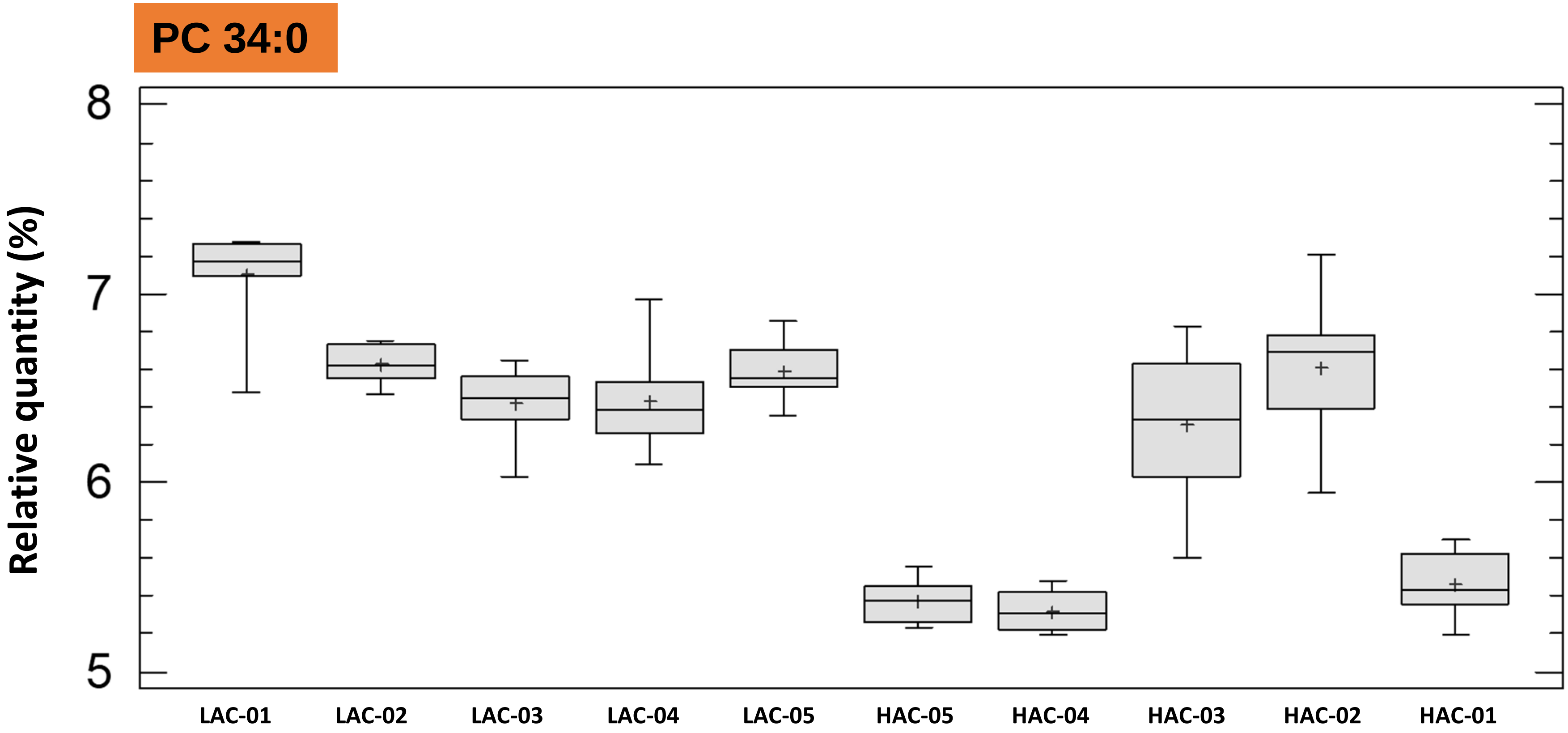

PC 34:0
8
7
6
5
Relative quantity (%)
LAC-01
LAC-02
LAC-03
LAC-04
LAC-05
HAC-05
HAC-04
HAC-03
HAC-02
HAC-01

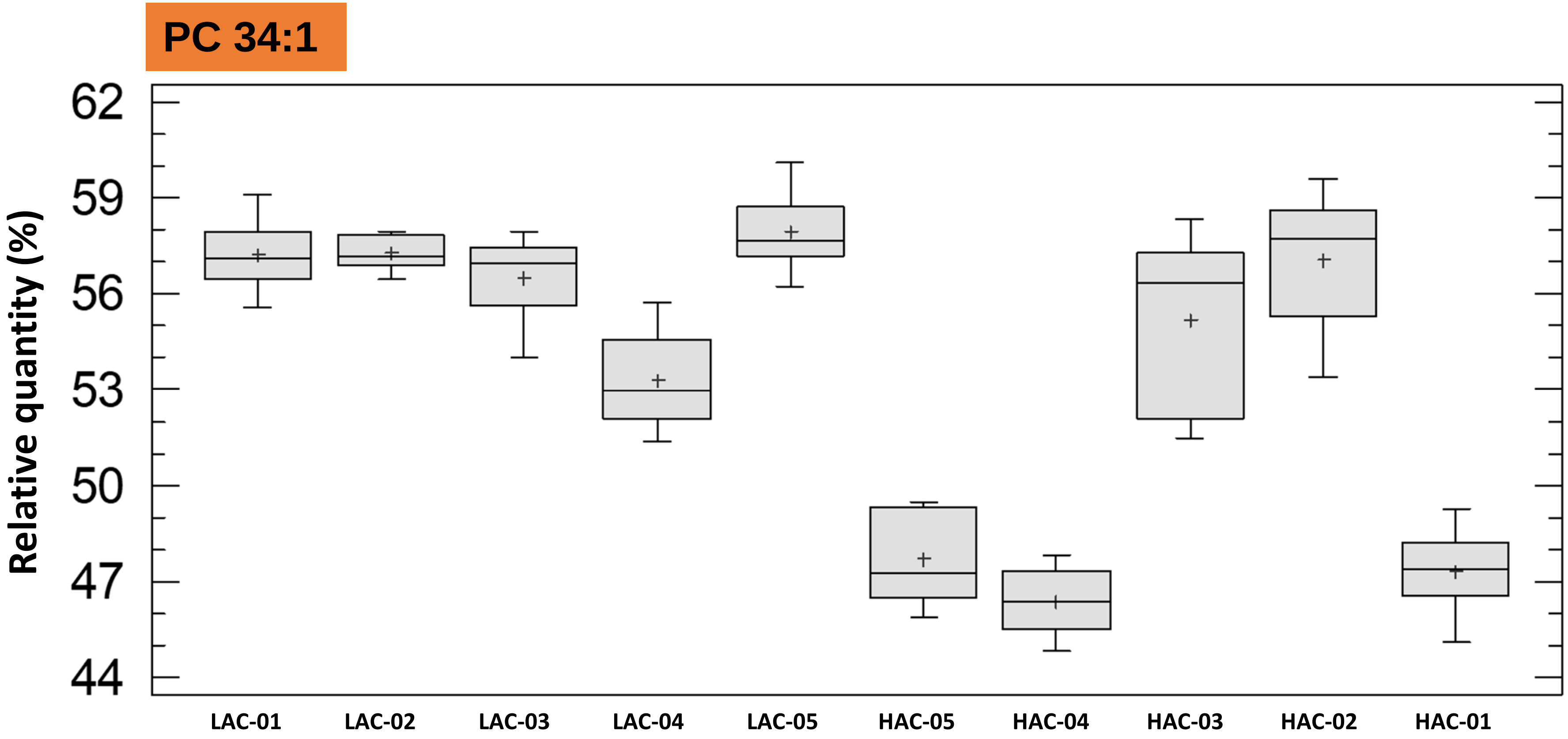

PC 34:1
Relative quantity (%)
62
59
56
53
50
47
44
LAC-01
LAC-02
LAC-03
LAC-04
LAC-05
HAC-05
HAC-04
HAC-03
HAC-02
HAC-01

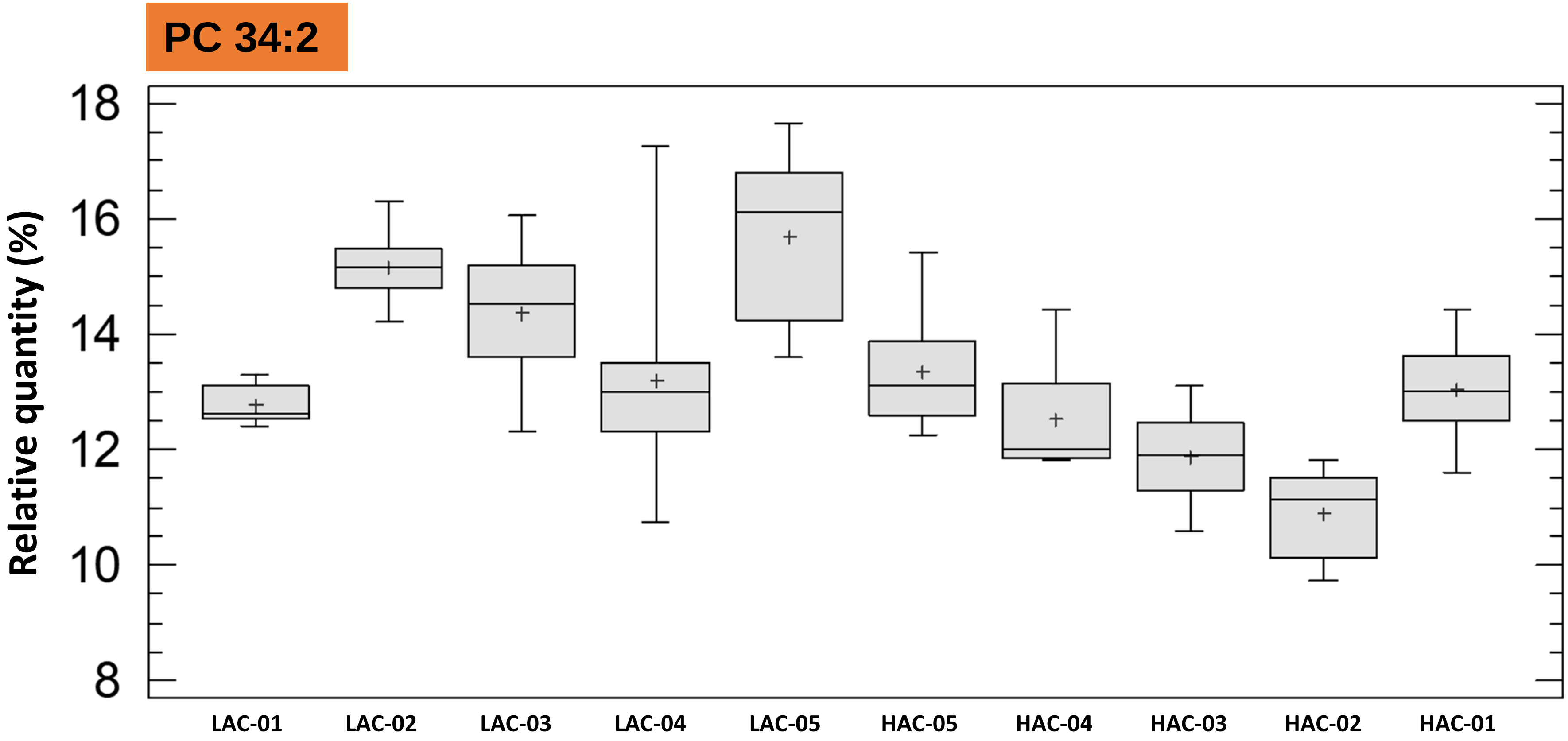

PC 34:2
Relative quantity (%)
18
16
14
12
10
8
LAC-01
LAC-02
LAC-03
LAC-04
LAC-05
HAC-05
HAC-04
HAC-03
HAC-02
HAC-01

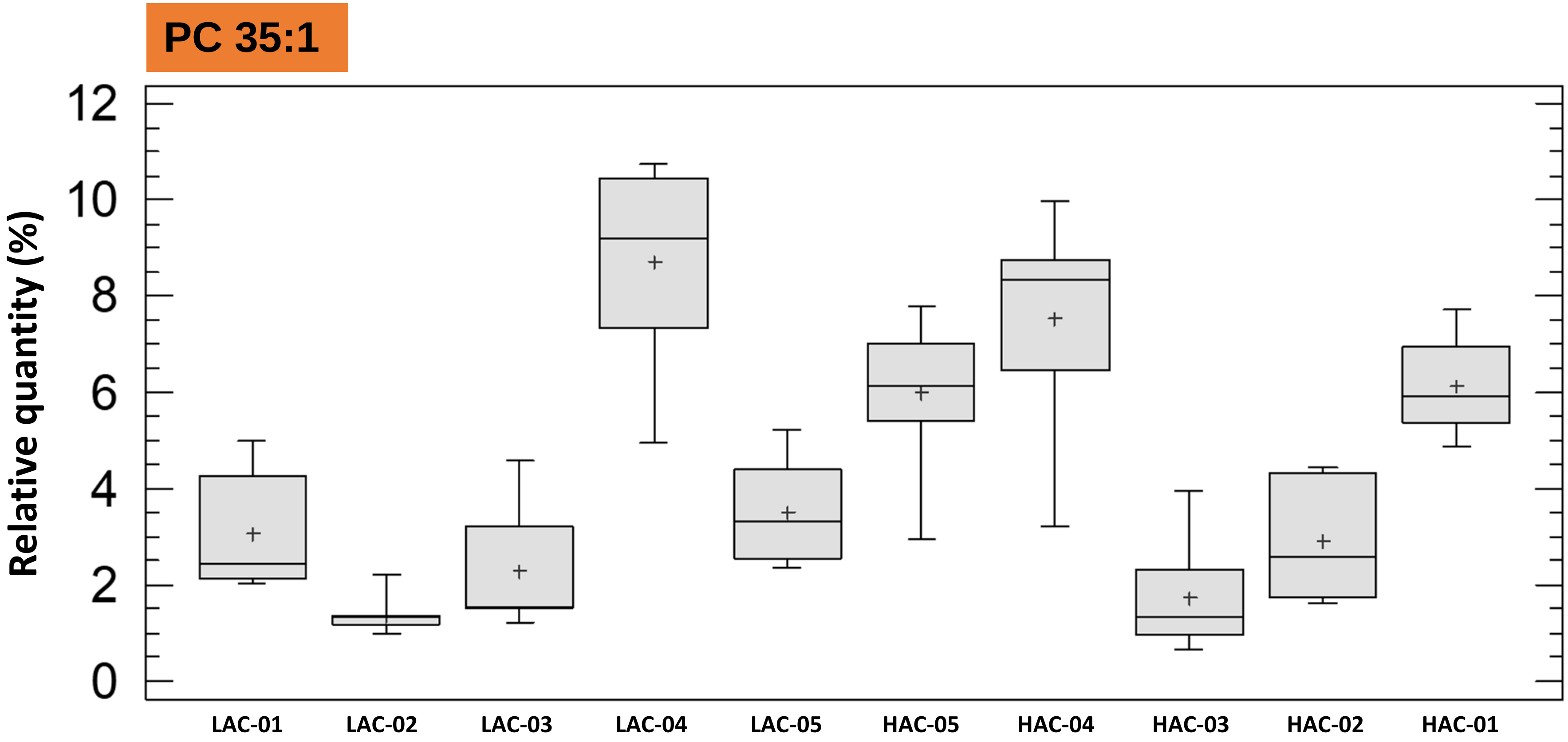

PC 35:1
Relative quantity (%)
0
2
4
6
8
10
12
LAC-01
LAC-02
LAC-03
LAC-04
LAC-05
HAC-05
HAC-04
HAC-03
HAC-02
HAC-01

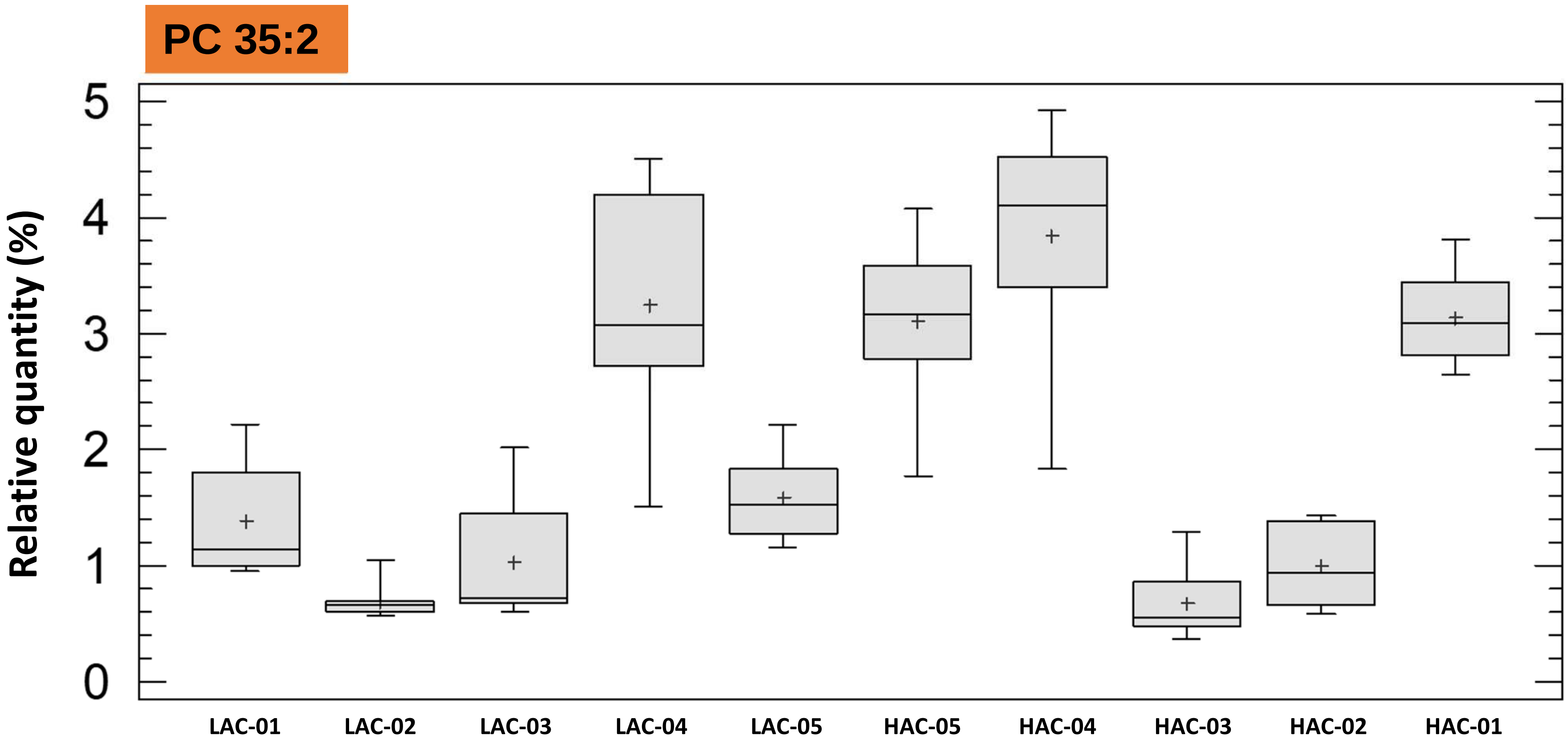

PC 35:2
Relative quantity (%)
0
1
2
3
4
5
LAC-01
LAC-02
LAC-03
LAC-04
LAC-05
HAC-05
HAC-04
HAC-03
HAC-02
HAC-01

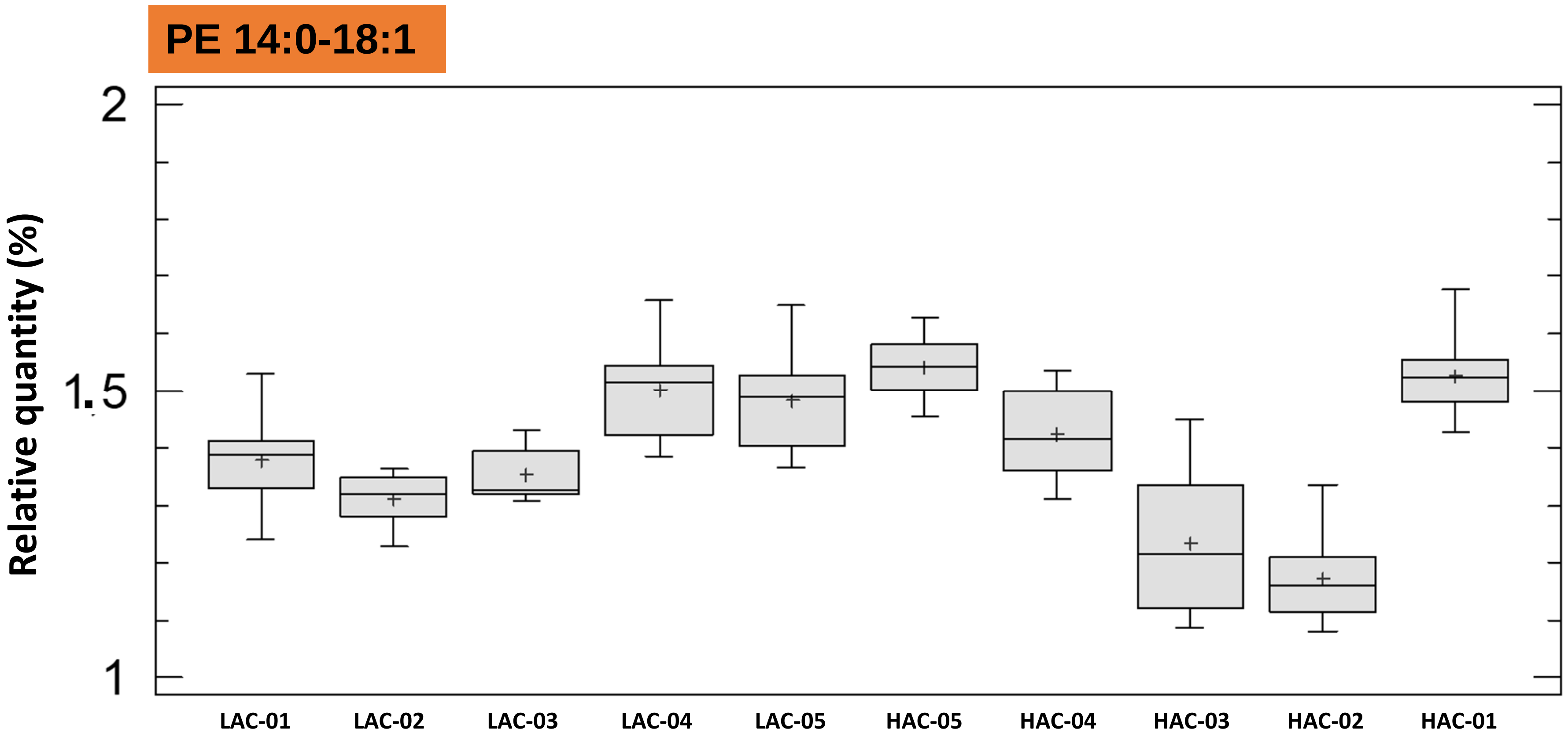
PE 14:0-18:1
2
1.5
1
Relative quantity (%)
LAC-01
LAC-02
LAC-03
LAC-04
LAC-05
HAC-05
HAC-04
HAC-03
HAC-02
HAC-01

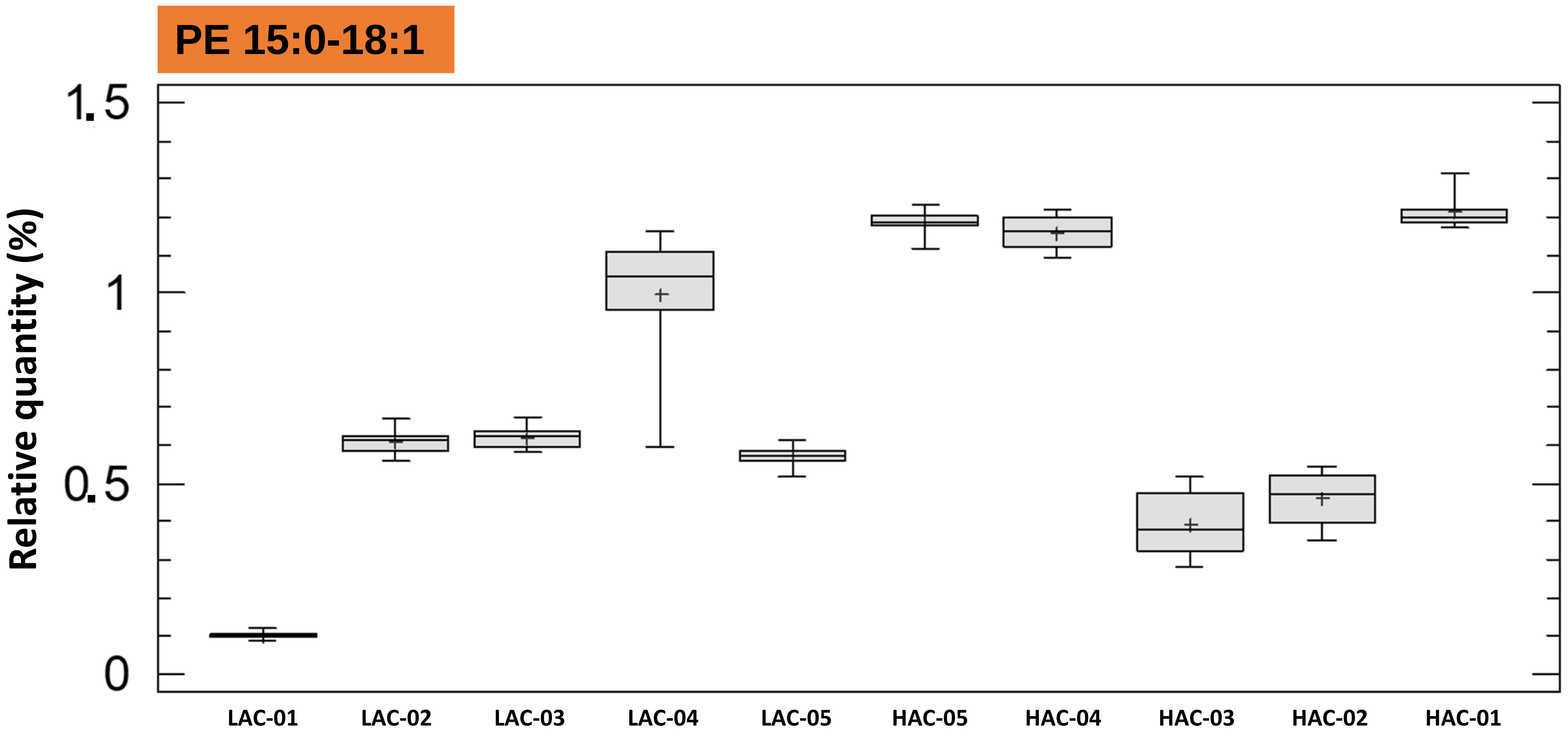

PE 15:0-18:1
Relative quantity (%)
1.5
1
0.5
0
LAC-01
LAC-02
LAC-03
LAC-04
LAC-05
HAC-05
HAC-04
HAC-03
HAC-02
HAC-01

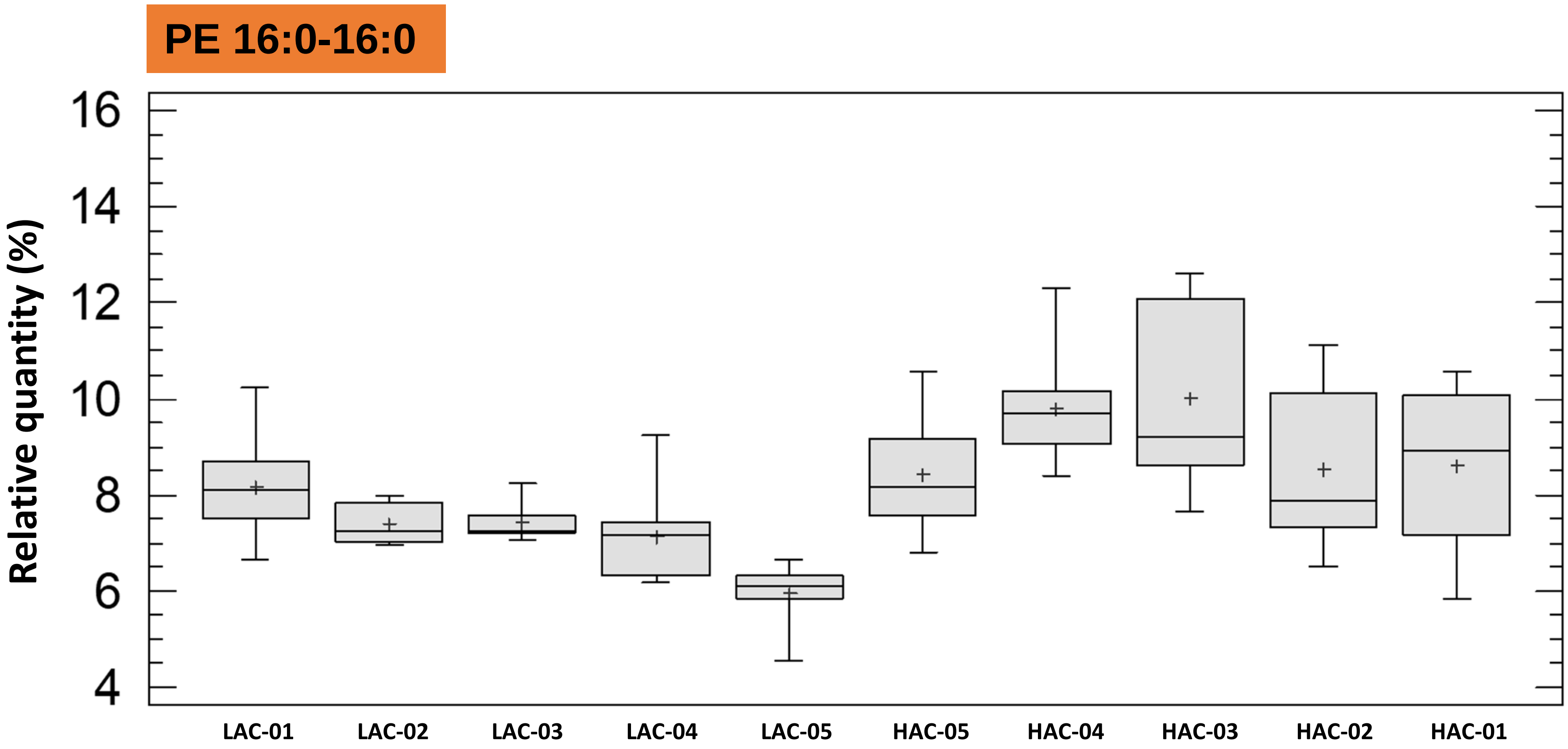

PE 16:0-16:0
Relative quantity (%)
16
14
12
10
8
6
4
LAC-01
LAC-02
LAC-03
LAC-04
LAC-05
HAC-05
HAC-04
HAC-03
HAC-02
HAC-01

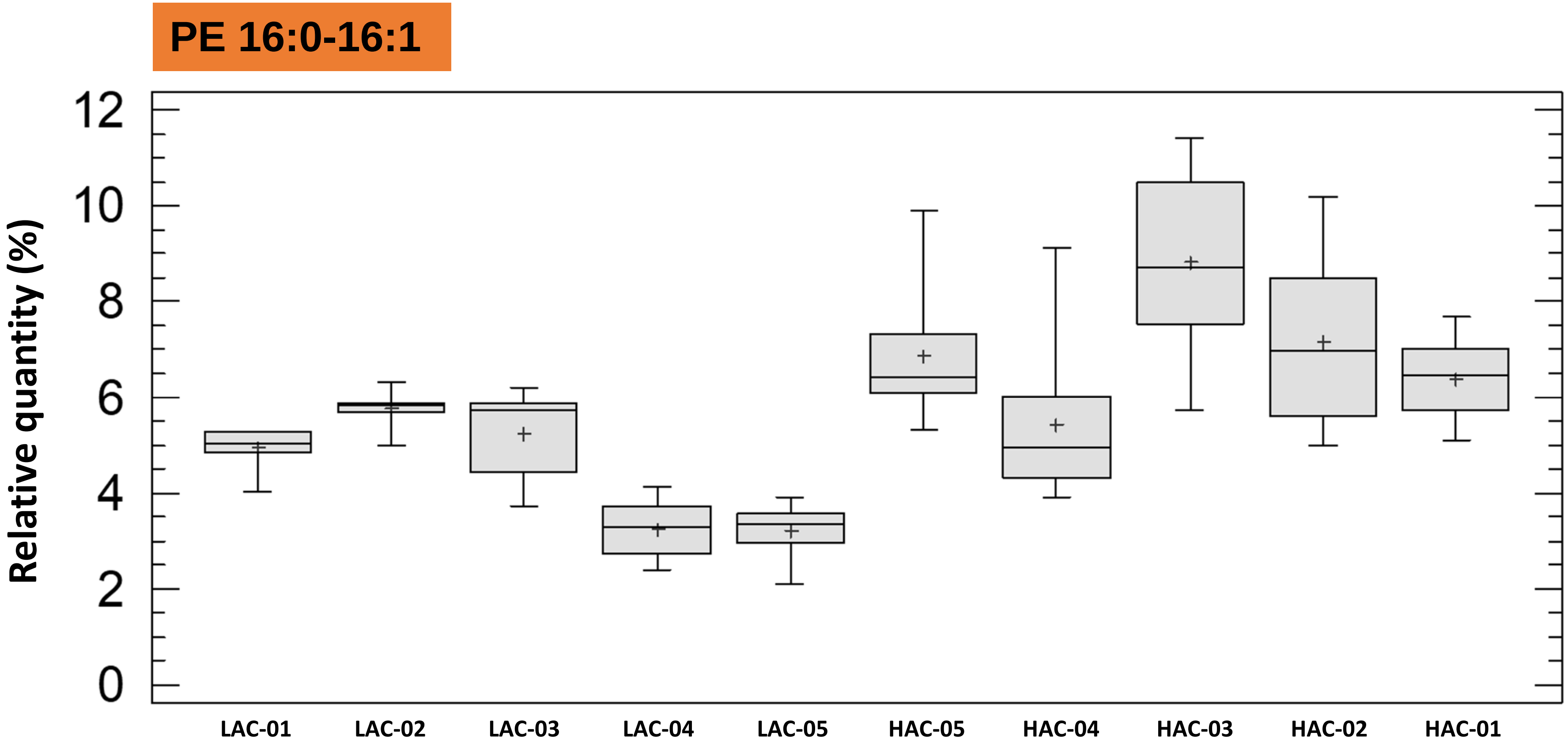
PE 16:0-16:1
Relative quantity (%)
0
2
4
6
8
10
12
LAC-01
LAC-02
LAC-03
LAC-04
LAC-05
HAC-05
HAC-04
HAC-03
HAC-02
HAC-01

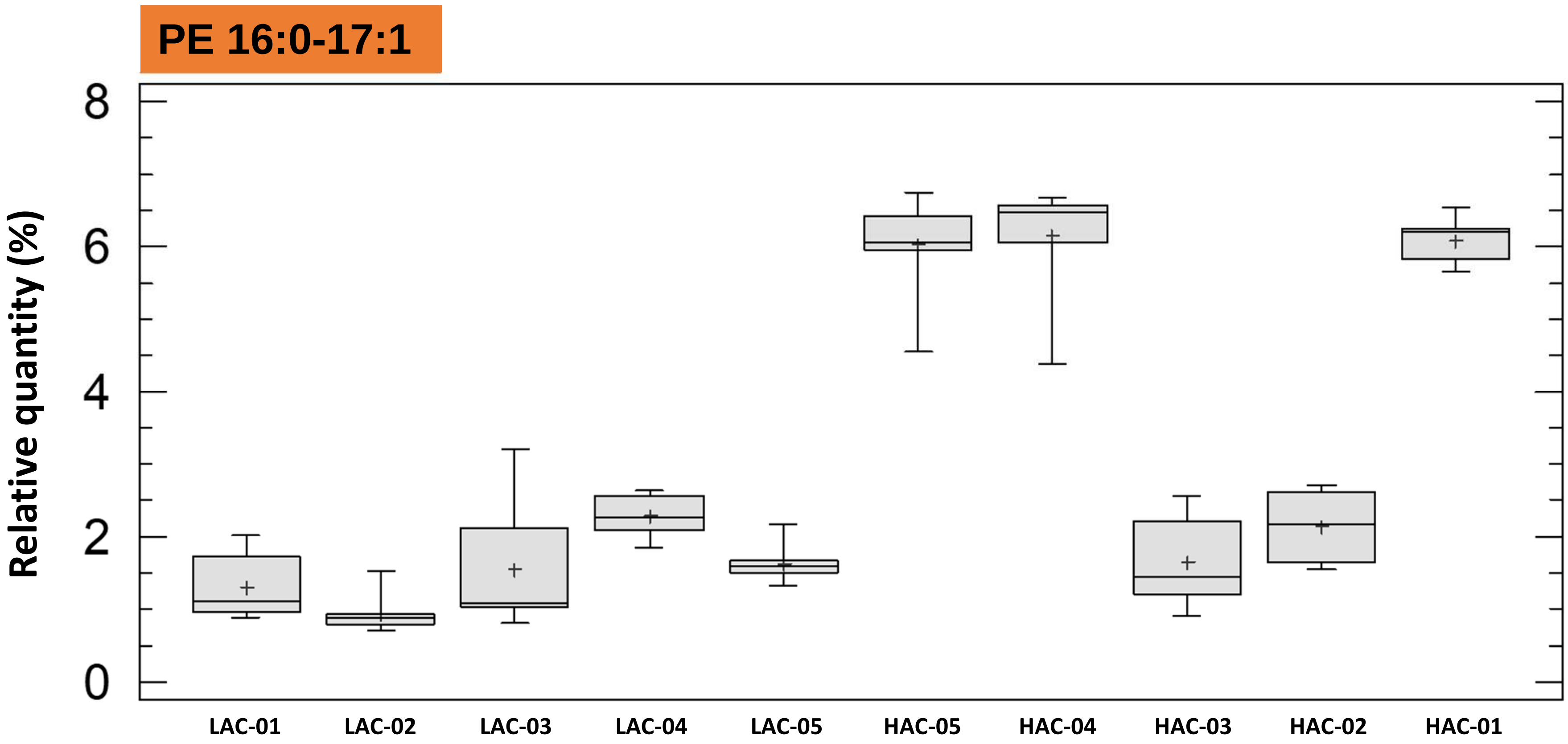
PE 16:0-17:1
Relative quantity (%)
0
2
4
6
8
LAC-01
LAC-02
LAC-03
LAC-04
LAC-05
HAC-05
HAC-04
HAC-03
HAC-02
HAC-01

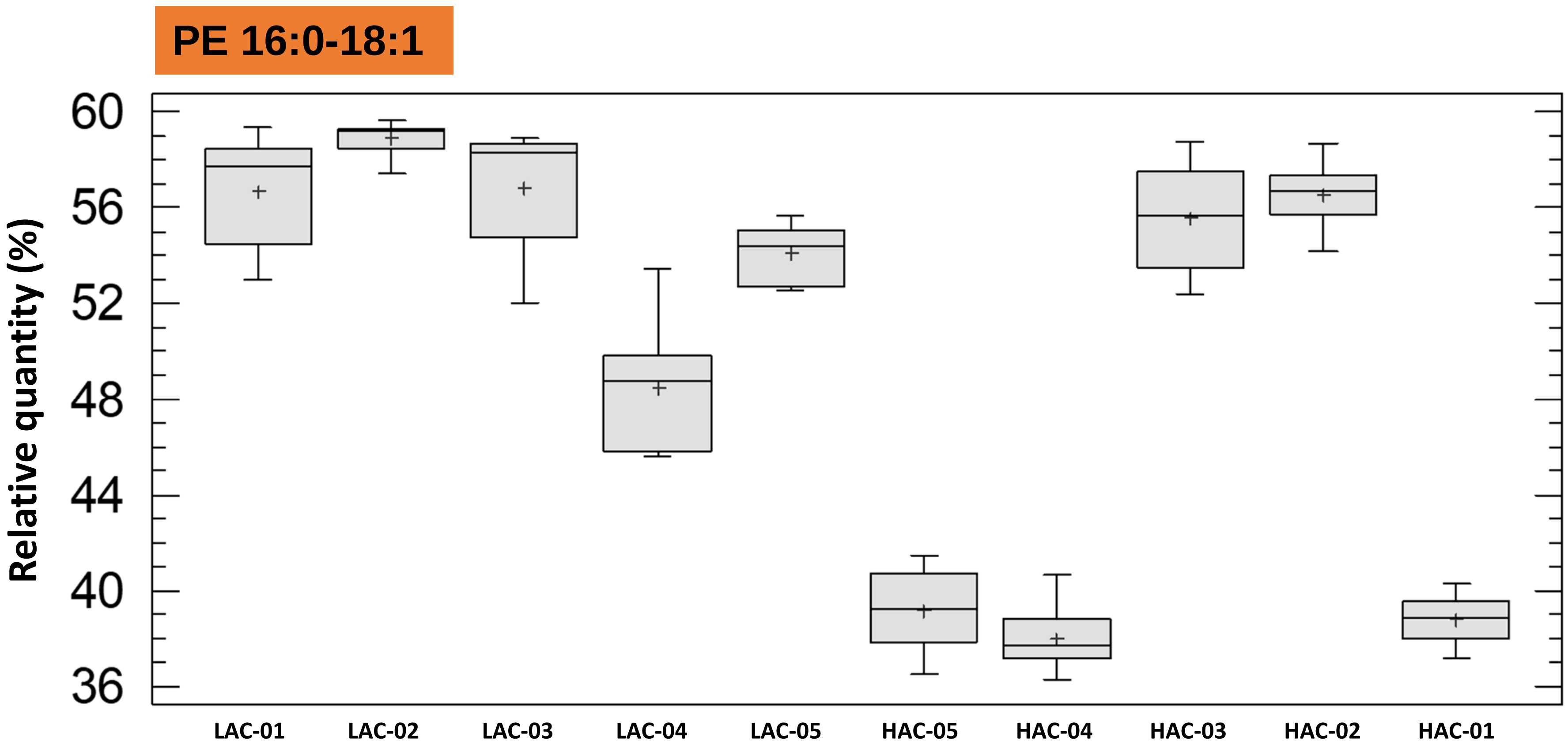

PE 16:0-18:1
Relative quantity (%)
60
56
52
48
44
40
36
LAC-01
LAC-02
LAC-03
LAC-04
LAC-05
HAC-05
HAC-04
HAC-03
HAC-02
HAC-01

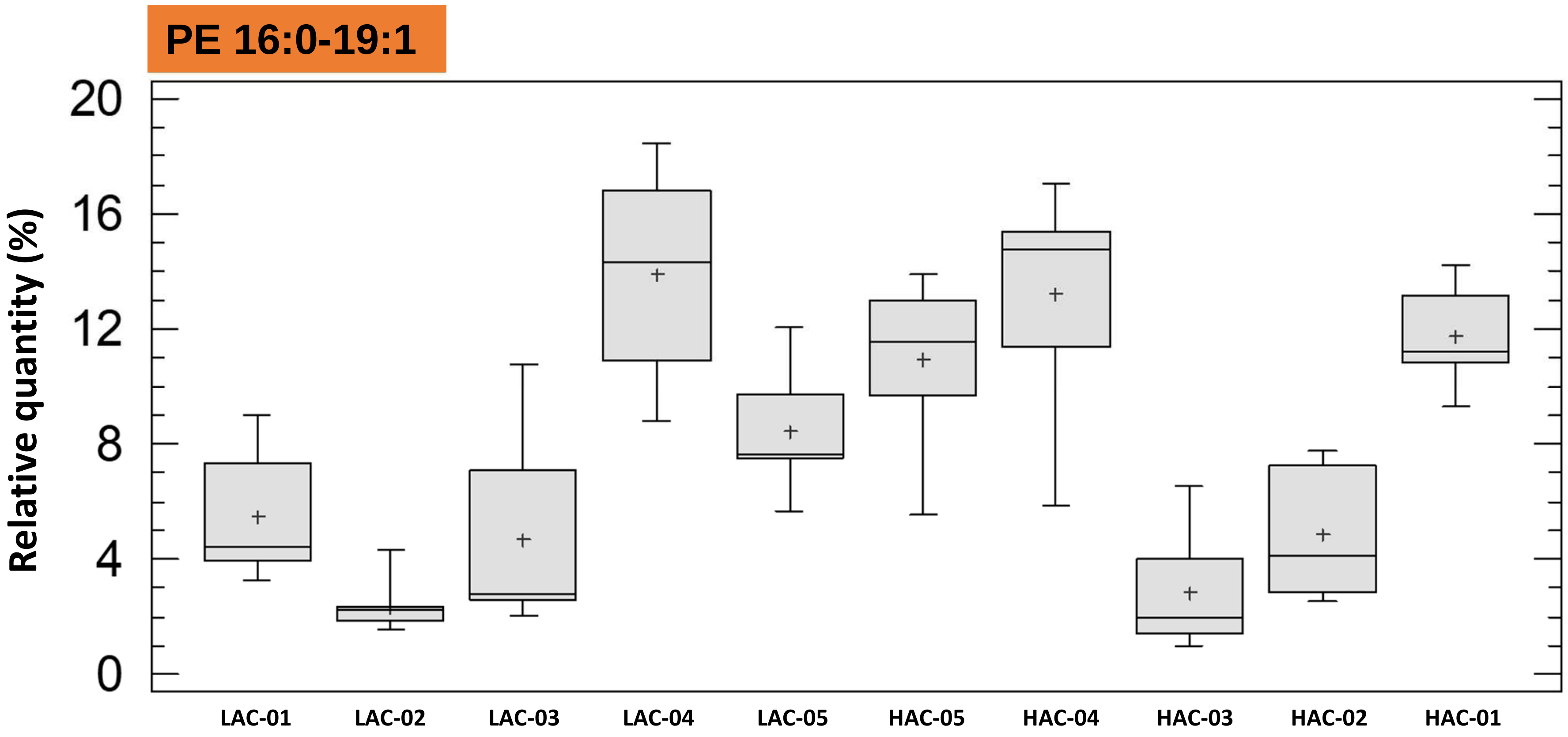
PE 16:0-19:1
Relative quantity (%)
20
16
12
8
4
0
LAC-01
LAC-02
LAC-03
LAC-04
LAC-05
HAC-05
HAC-04
HAC-03
HAC-02
HAC-01

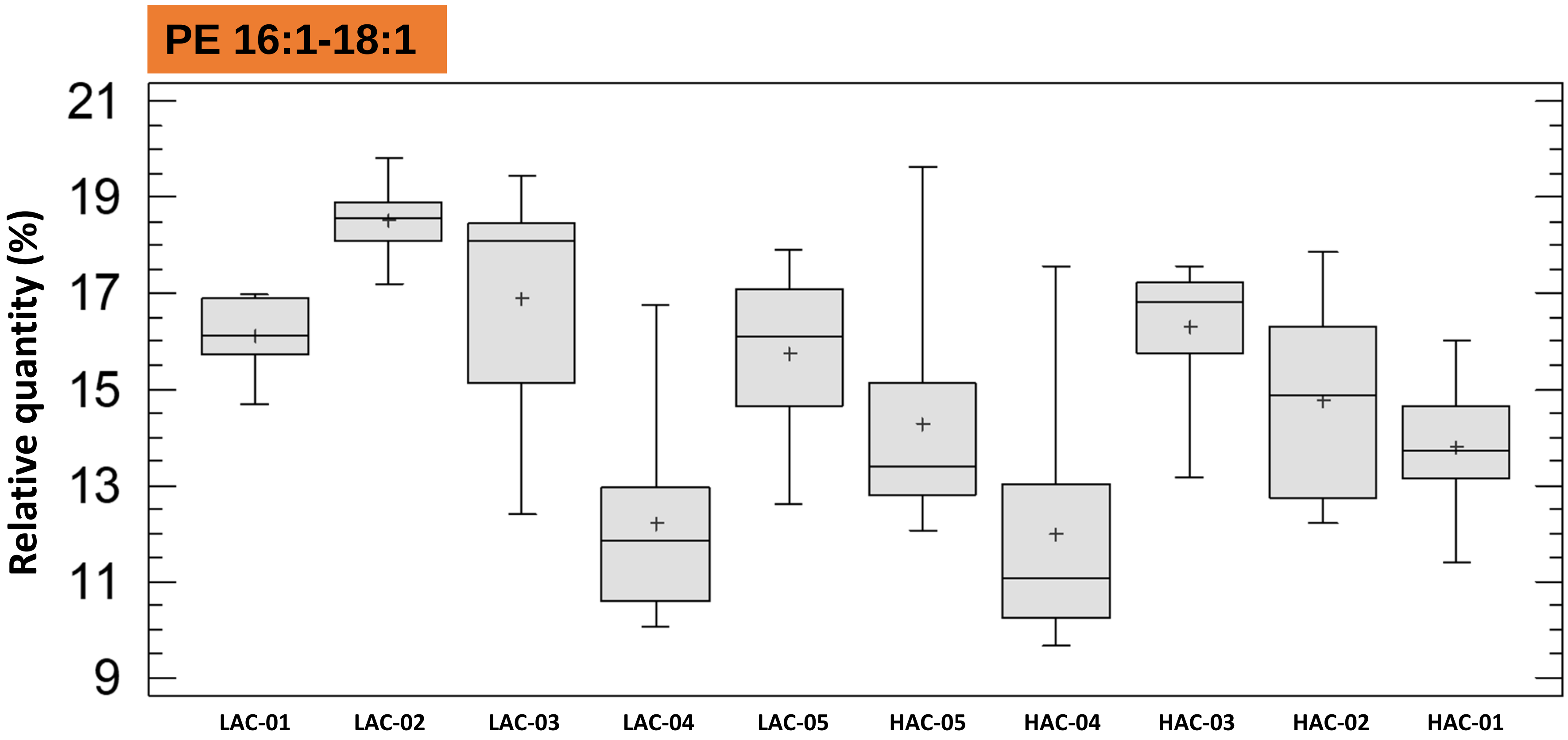
PE 16:1-18:1
Relative quantity (%)
21
19
17
15
13
11
9
LAC-01
LAC-02
LAC-03
LAC-04
LAC-05
HAC-05
HAC-04
HAC-03
HAC-02
HAC-01

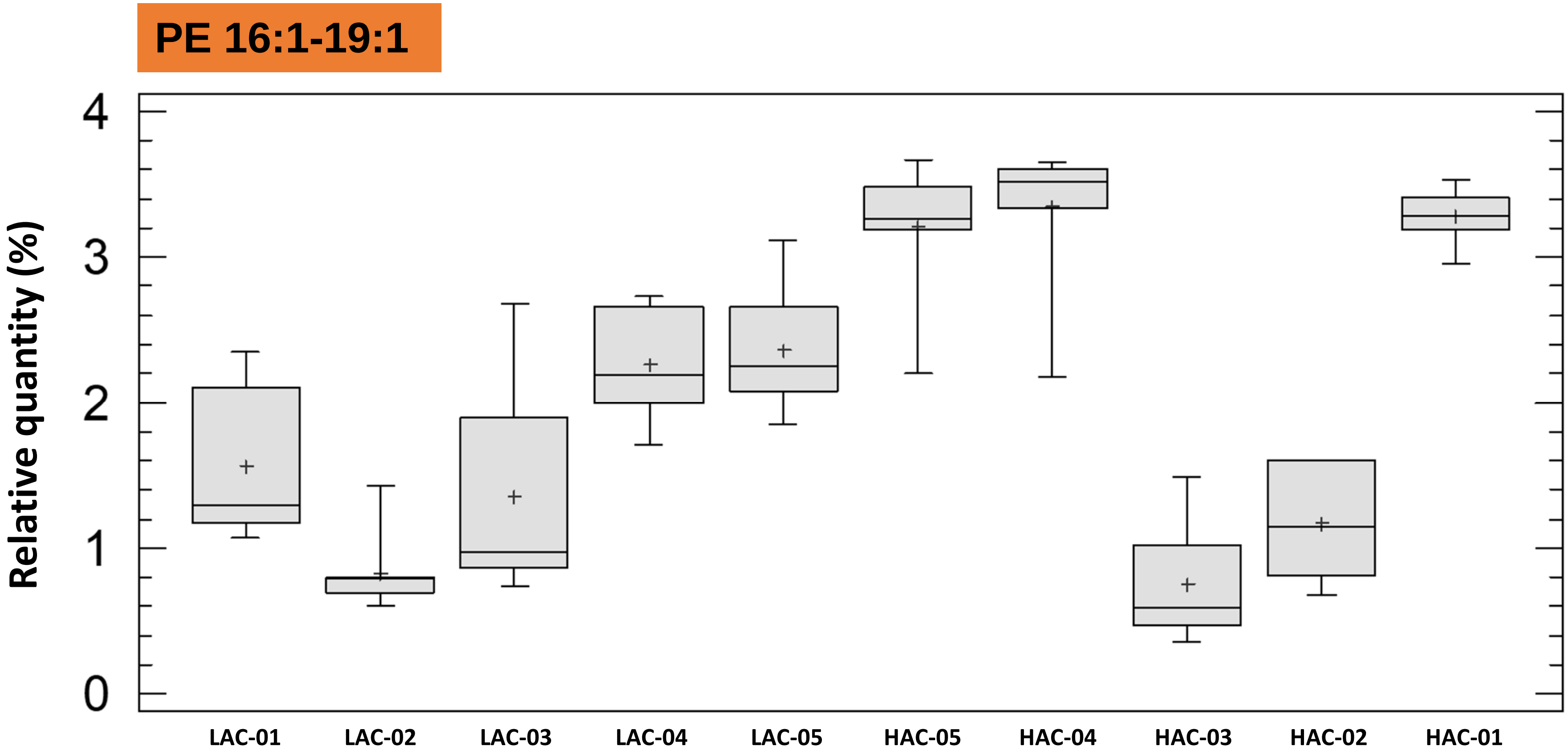
PE 16:1-19:1
Relative quantity (%)
4
3
2
1
0
LAC-01
LAC-02
LAC-03
LAC-04
LAC-05
HAC-05
HAC-04
HAC-03
HAC-02
HAC-01

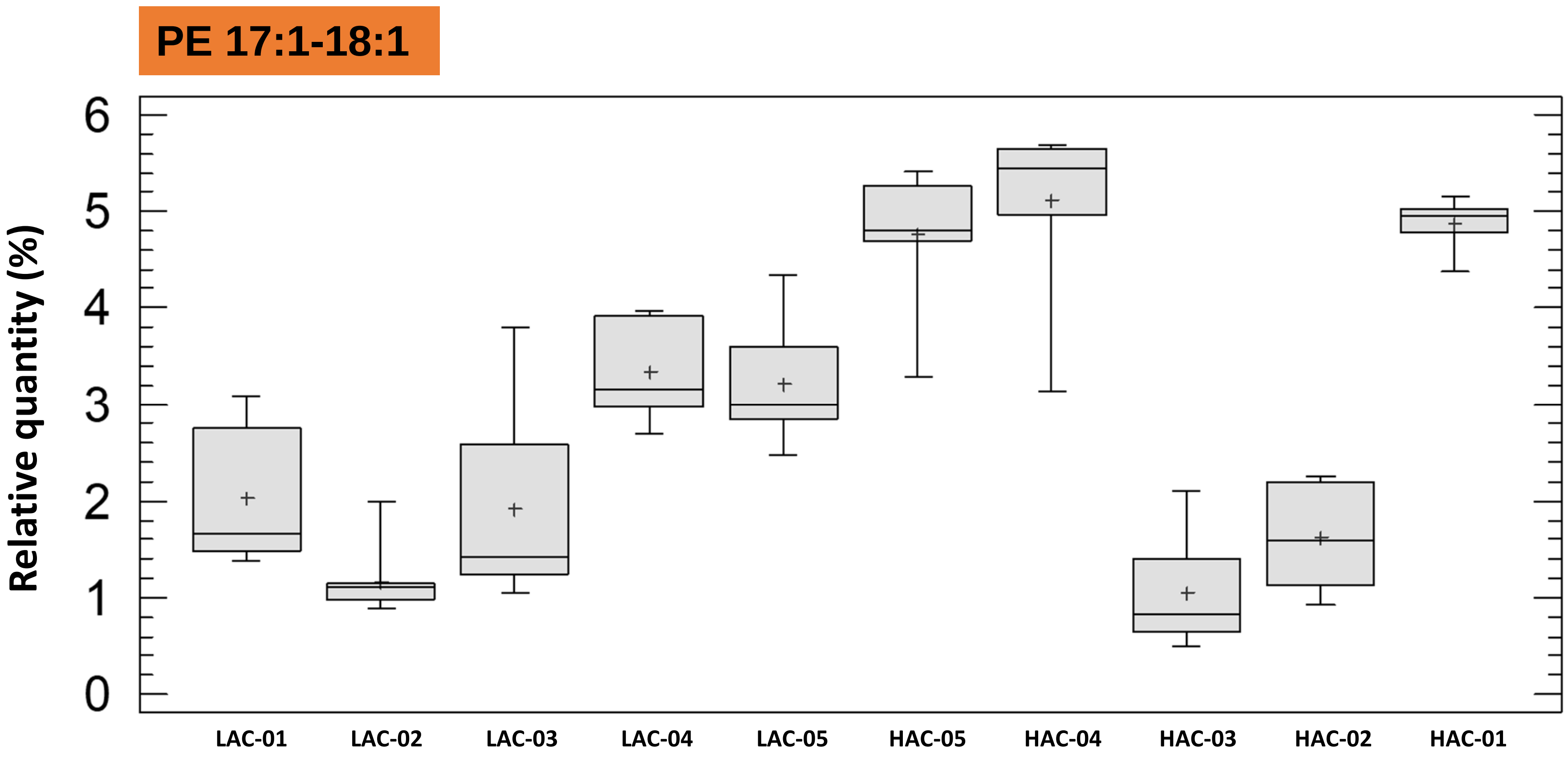

PE 17:1-18:1
Relative quantity (%)
0
1
2
3
4
5
6
LAC-01
LAC-02
LAC-03
LAC-04
LAC-05
HAC-05
HAC-04
HAC-03
HAC-02
HAC-01

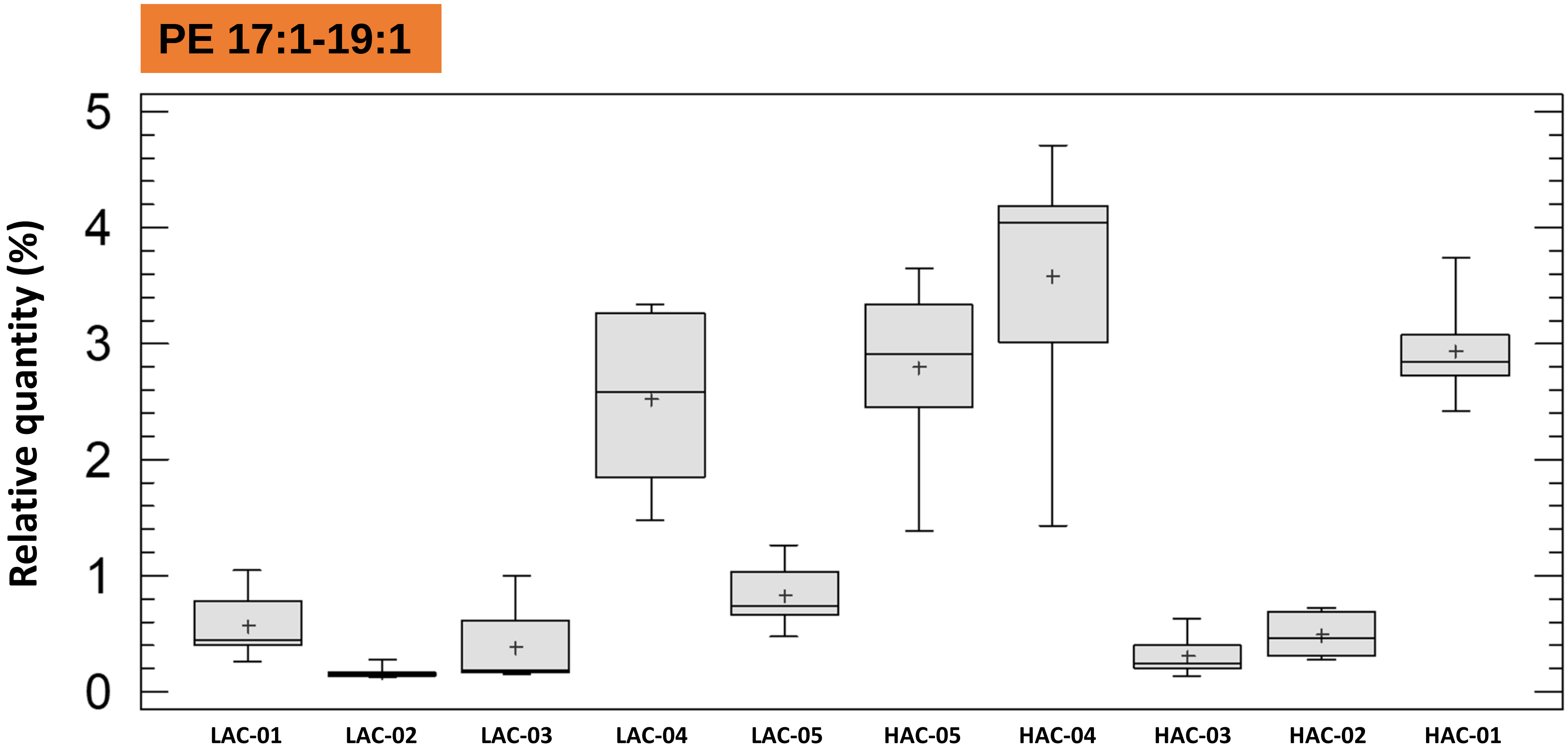
PE 17:1-19:1
Relative quantity (%)
0
1
2
3
4
5
LAC-01
LAC-02
LAC-03
LAC-04
LAC-05
HAC-05
HAC-04
HAC-03
HAC-02
HAC-01

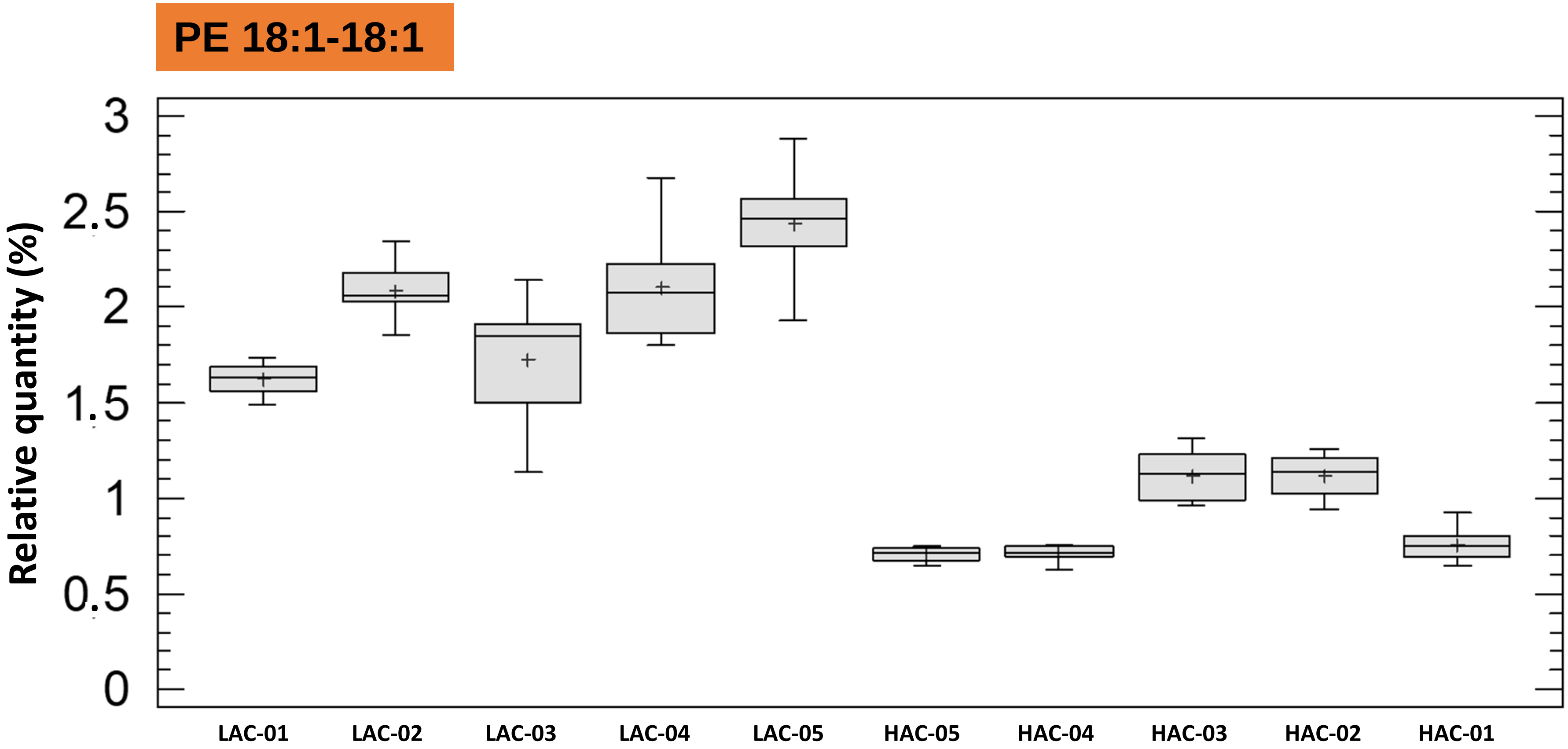
PE 18:1-18:1
Relative quantity (%)
3
2.5
2
1.5
1
0.5
0
LAC-01
LAC-02
LAC-03
LAC-04
LAC-05
HAC-05
HAC-04
HAC-03
HAC-02
HAC-01

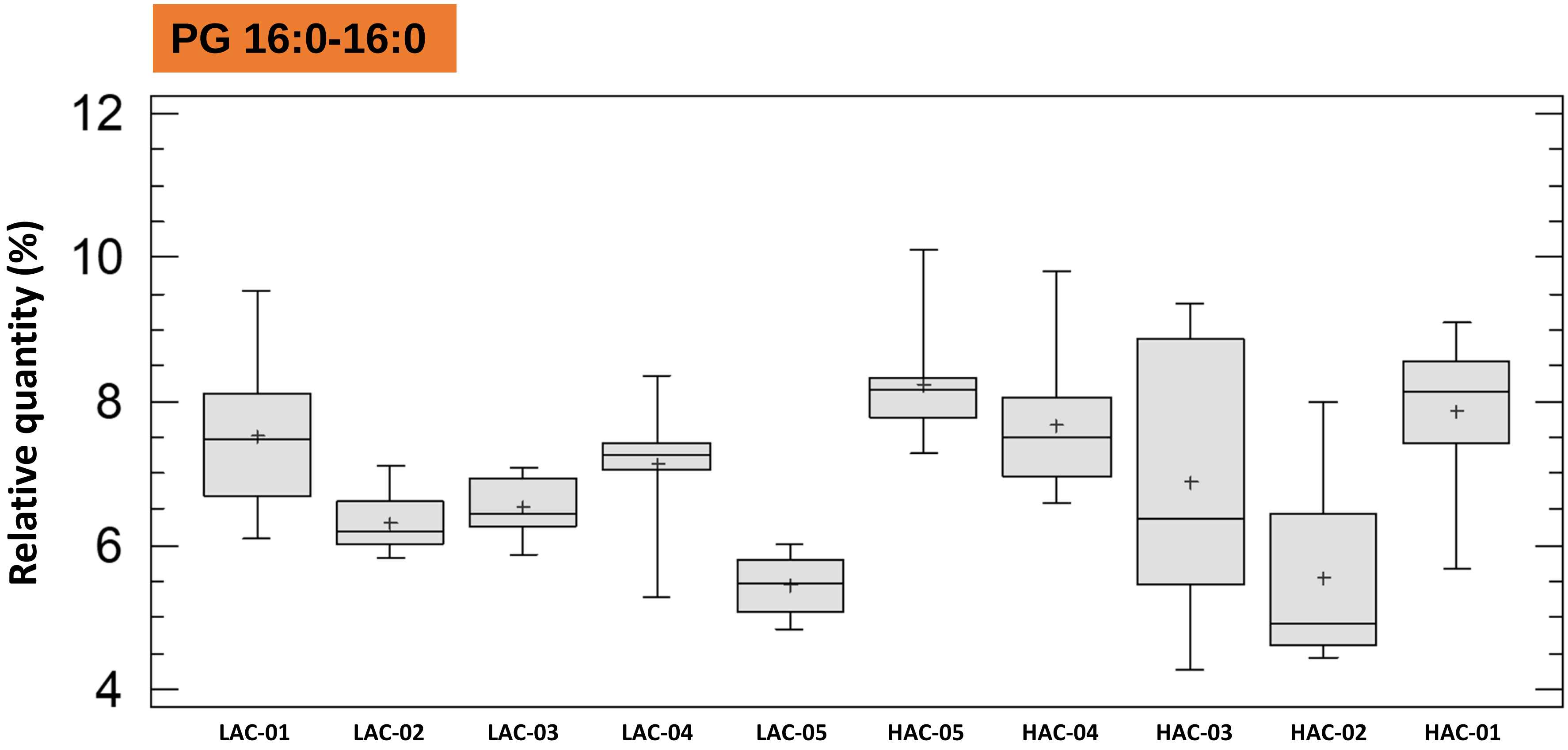
PG 16:0-16:0
Relative quantity (%)
12
10
8
6
4
LAC-01
LAC-02
LAC-03
LAC-04
LAC-05
HAC-05
HAC-04
HAC-03
HAC-02
HAC-01

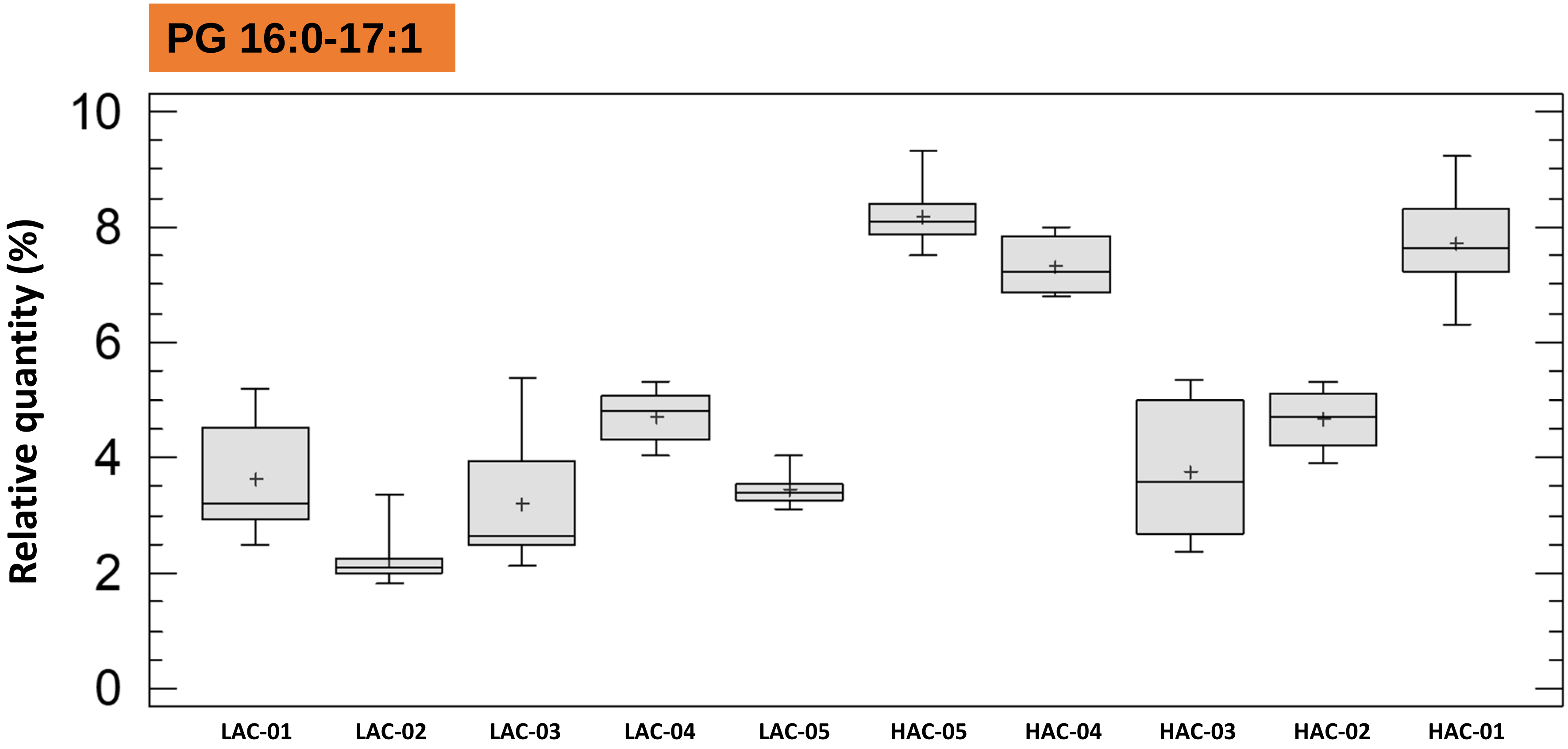

PG 16:0-17:1
Relative quantity (%)
10
8
6
4
2
0
LAC-01
LAC-02
LAC-03
LAC-04
LAC-05
HAC-05
HAC-04
HAC-03
HAC-02
HAC-01

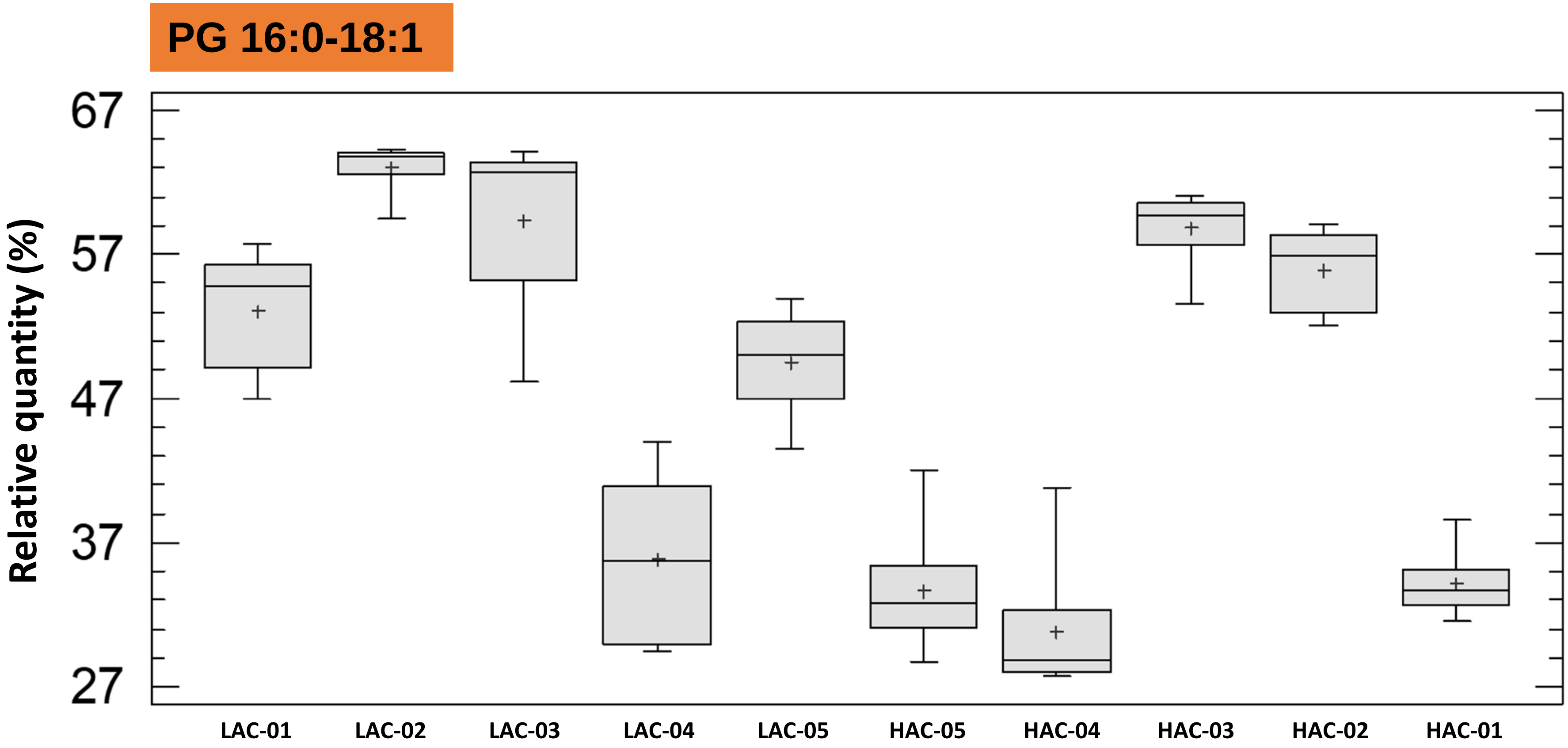
PG 16:0-18:1
Relative quantity (%)
67
57
47
37
27
LAC-01
LAC-02
LAC-03
LAC-04
LAC-05
HAC-05
HAC-04
HAC-03
HAC-02
HAC-01

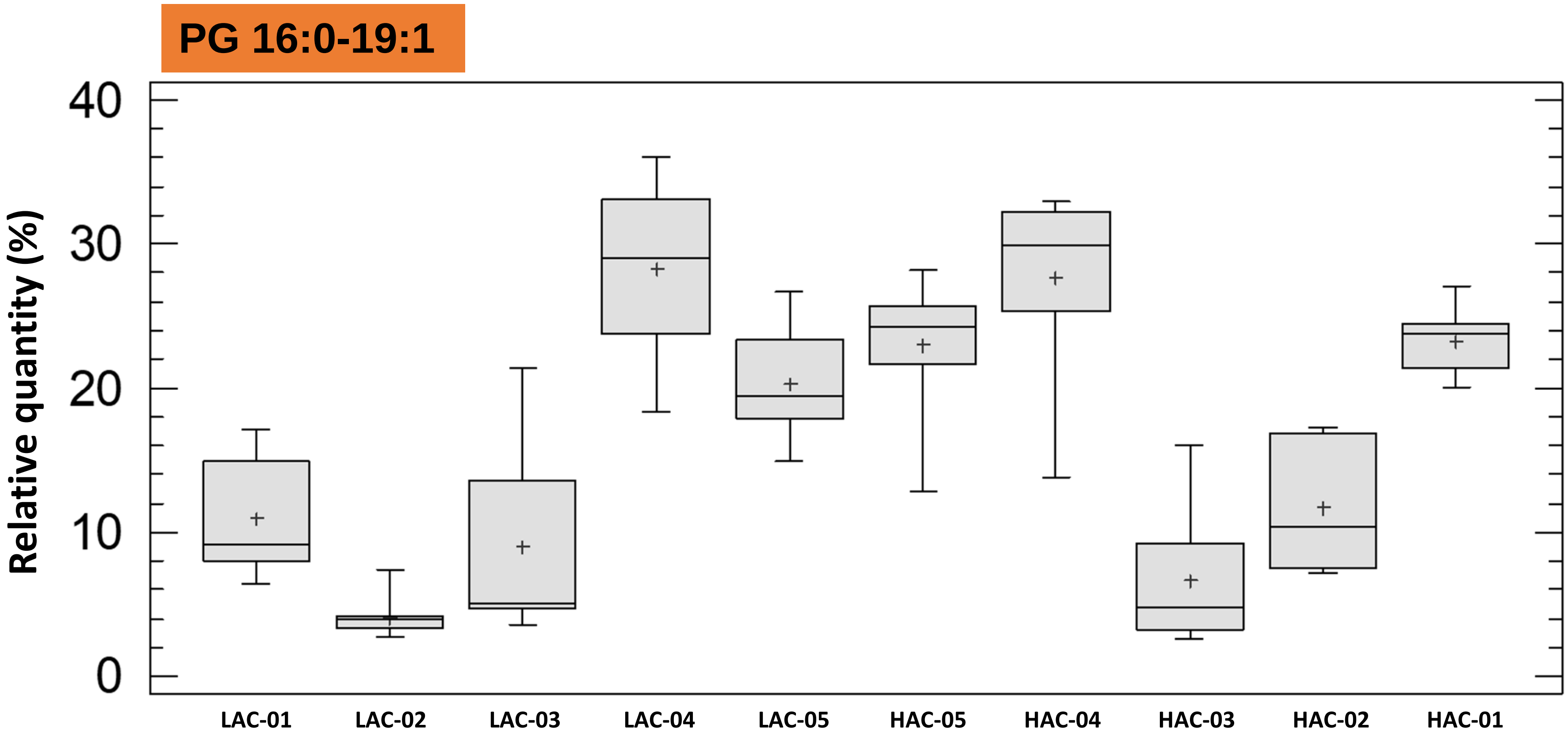
PG 16:0-19:1
Relative quantity (%)
0
10
20
30
40
LAC-01
LAC-02
LAC-03
LAC-04
LAC-05
HAC-05
HAC-04
HAC-03
HAC-02
HAC-01

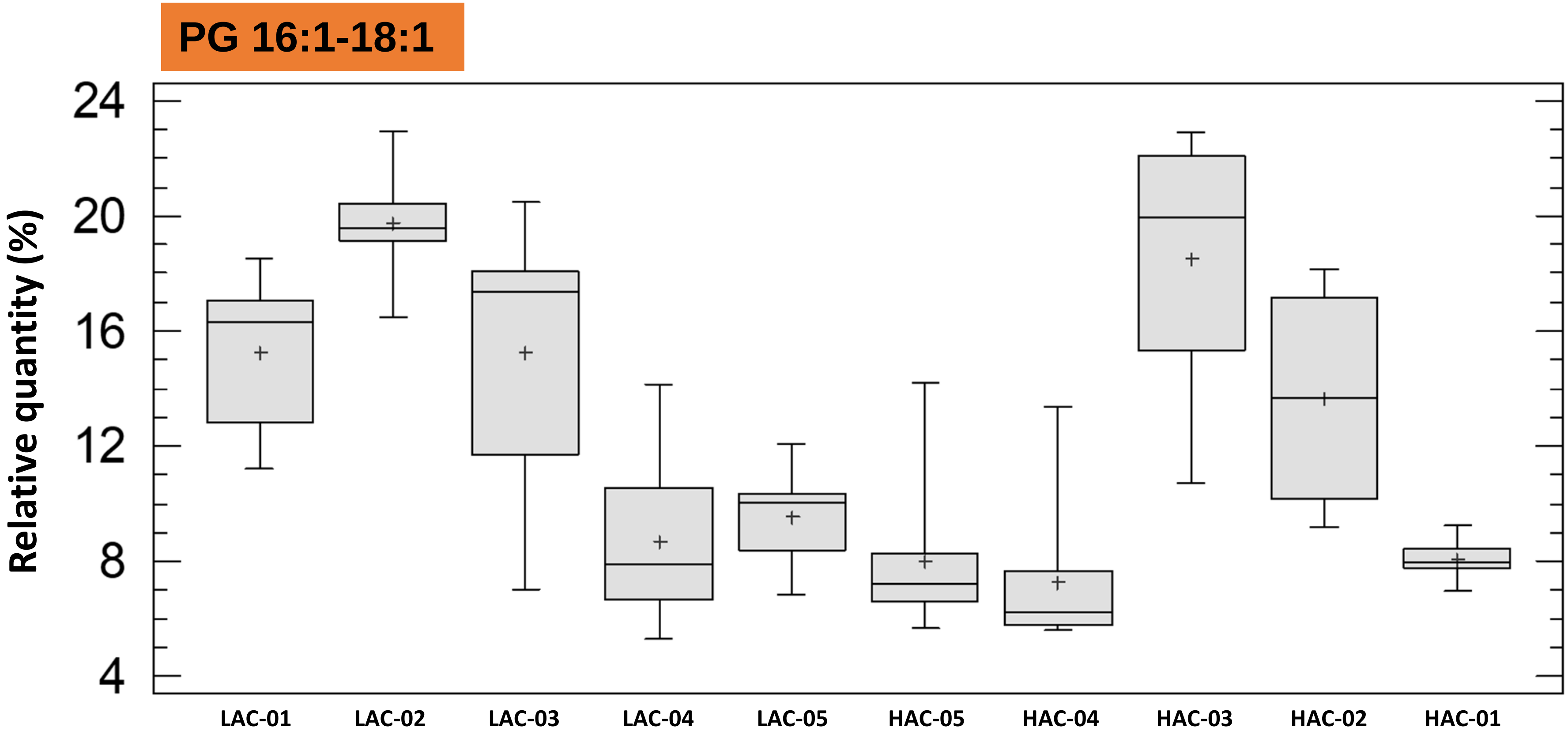

PG 16:1-18:1
Relative quantity (%)
24
20
16
12
8
4
LAC-01
LAC-02
LAC-03
LAC-04
LAC-05
HAC-05
HAC-04
HAC-03
HAC-02
HAC-01

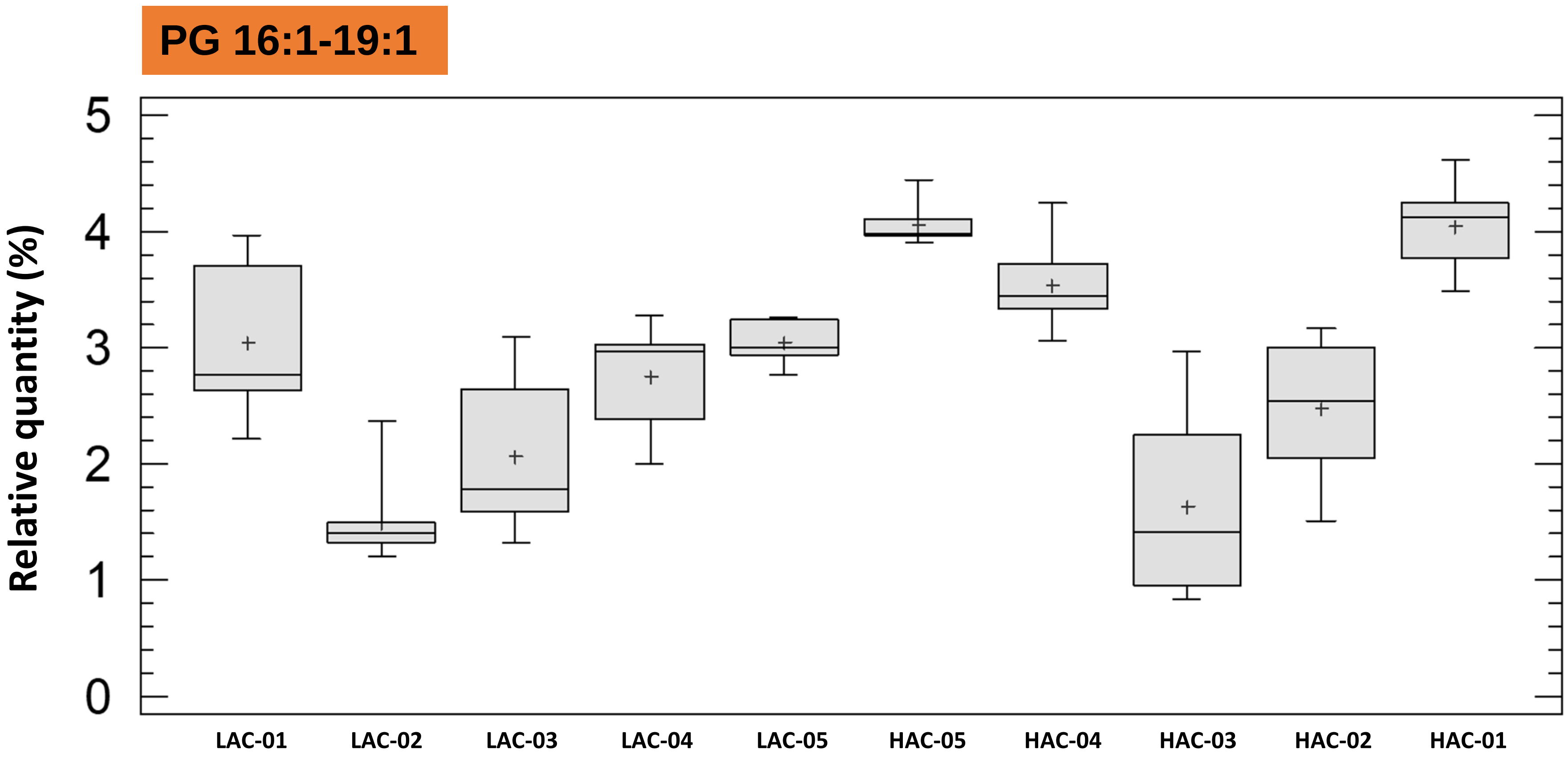
PG 16:1-19:1
Relative quantity (%)
0
1
2
3
4
5
LAC-01
LAC-02
LAC-03
LAC-04
LAC-05
HAC-05
HAC-04
HAC-03
HAC-02
HAC-01

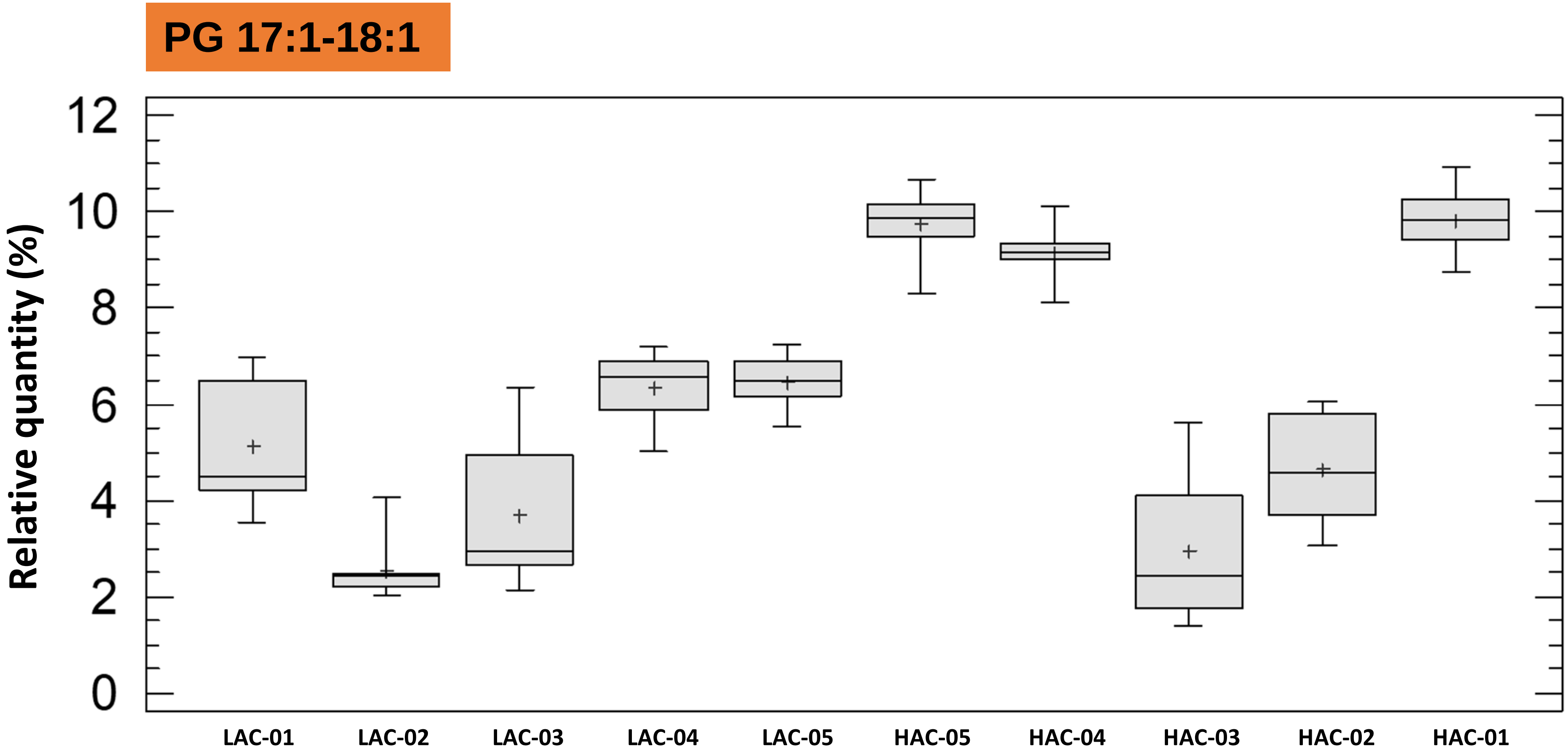
PG 17:1-18:1
Relative quantity (%)
0
2
4
6
8
10
12
LAC-01
LAC-02
LAC-03
LAC-04
LAC-05
HAC-05
HAC-04
HAC-03
HAC-02
HAC-01

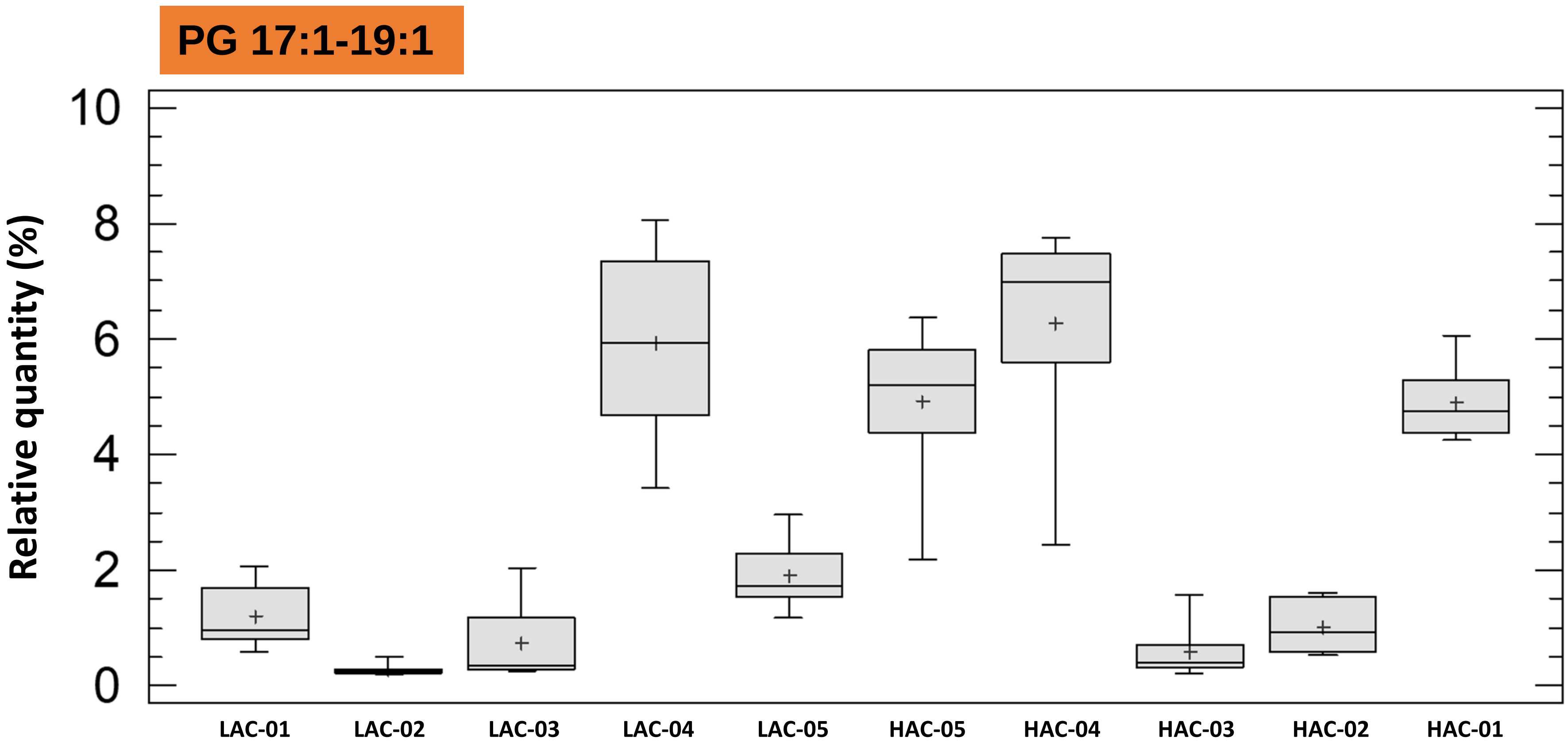
PG 17:1-19:1
Relative quantity (%)
10
8
6
4
2
0
LAC-01
LAC-02
LAC-03
LAC-04
LAC-05
HAC-05
HAC-04
HAC-03
HAC-02
HAC-01

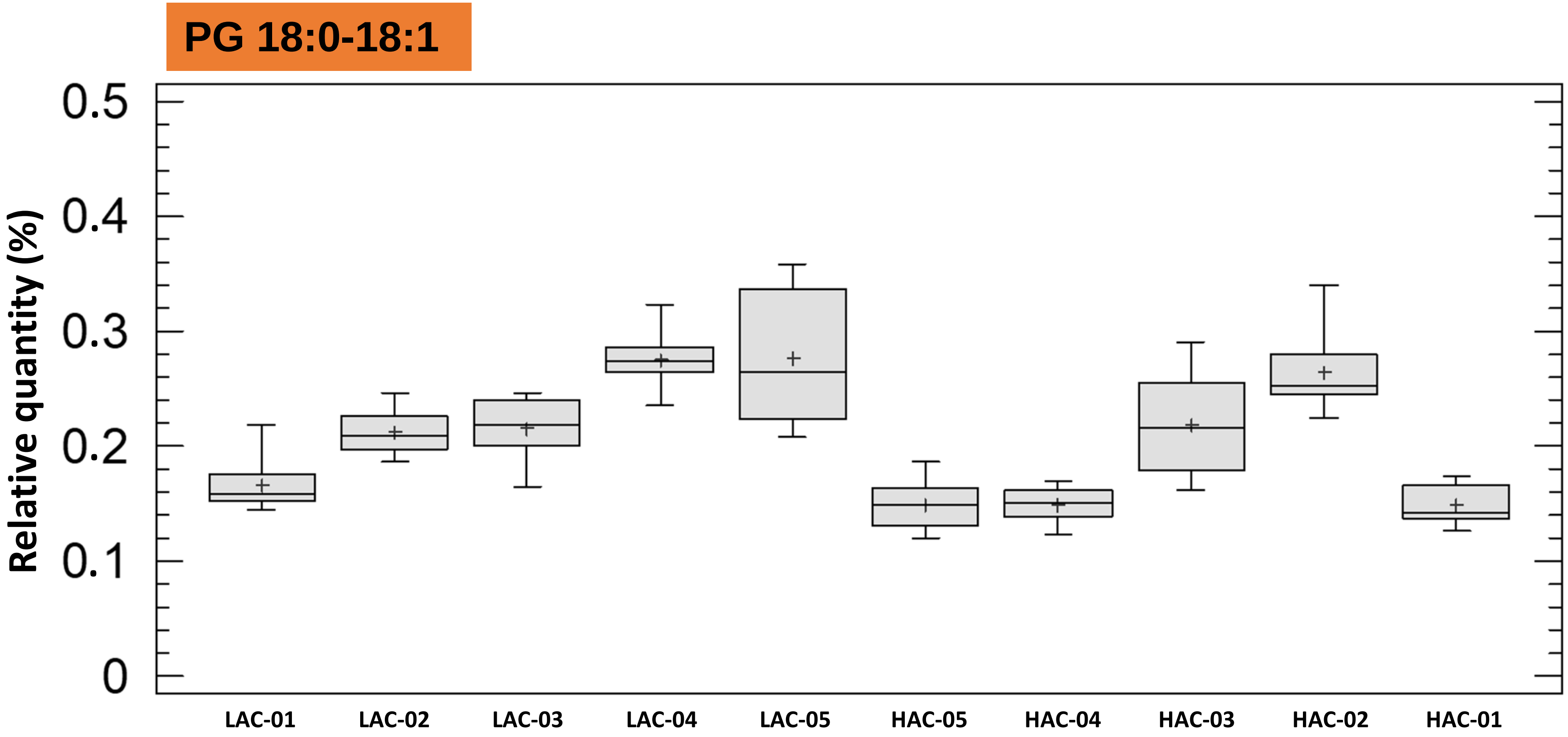

PG 18:0-18:1
Relative quantity (%)
0.5
0.4
0.3
0.2
0.1
0
LAC-01
LAC-02
LAC-03
LAC-04
LAC-05
HAC-05
HAC-04
HAC-03
HAC-02
HAC-01
